# Triplet Upconversion under Ambient Conditions Enables Digital Light Processing 3D Printing

**DOI:** 10.1021/acscentsci.3c01263

**Published:** 2024-01-16

**Authors:** Connor J. O’Dea, Jussi Isokuortti, Emma E. Comer, Sean T. Roberts, Zachariah A. Page

**Affiliations:** Department of Chemistry, The University of Texas at Austin, Austin, Texas 78712 ,United States

## Abstract

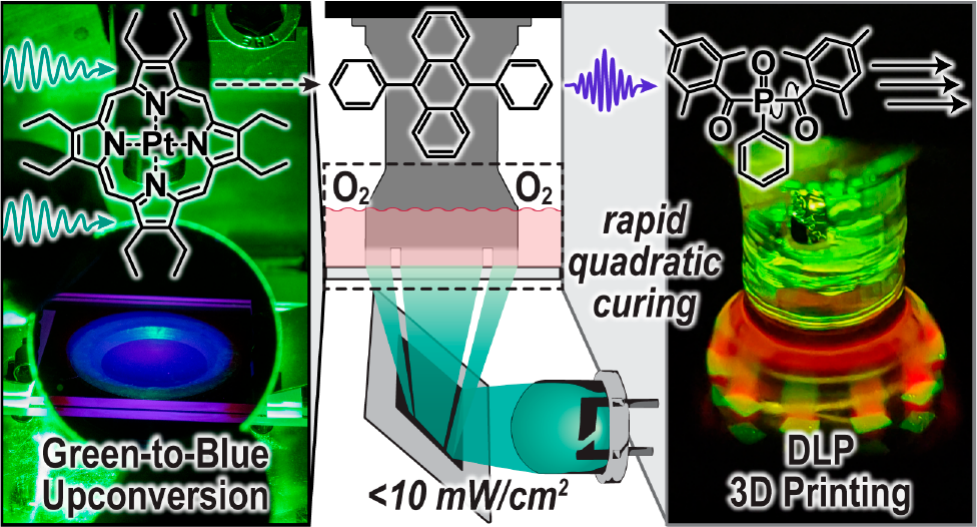

The rapid photochemical conversion of materials from liquid to solid (i.e., curing) has enabled the fabrication of modern plastics used in microelectronics, dentistry, and medicine. However, industrialized photocurables remain restricted to unimolecular bond homolysis reactions (Type I photoinitiations) that are driven by high-energy UV light. This narrow mechanistic scope both challenges the production of high-resolution objects and restricts the materials that can be produced using emergent manufacturing technologies (e.g., 3D printing). Herein we develop a photosystem based on triplet–triplet annihilation upconversion (TTA-UC) that efficiently drives a Type I photocuring process using green light at low power density (<10 mW/cm^2^) and in the presence of ambient oxygen. This system also exhibits a superlinear dependence of its cure depth on the light exposure intensity, which enhances spatial resolution. This enables for the first-time integration of TTA-UC in an inexpensive, rapid, and high-resolution manufacturing process, digital light processing (DLP) 3D printing. Moreover, relative to traditional Type I and Type II (photoredox) strategies, the present TTA-UC photoinitiation method results in improved cure depth confinement and resin shelf stability. This report provides a user-friendly avenue to utilize TTA-UC in ambient photochemical processes and paves the way toward fabrication of next-generation plastics with improved geometric precision and functionality.

## Abstract 2

Converting green light to violet light within a vat of liquid resin enables printing of high-resolution 3D structures under air.

## Introduction

**Figure 1:**
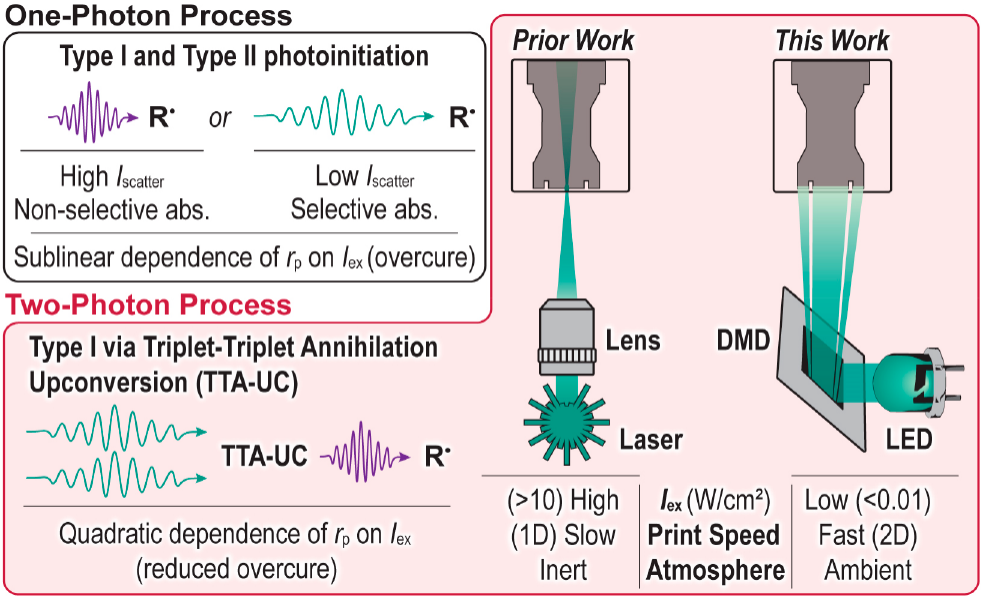
Previous work based on one- and two-photon stereolithographic methods vs this work applying TTA-UC for two-photon digital light processing 3D printing. *I*_scatter_, scattered light intensity; abs., absorbance; *r*_p_, rate of polymerization; *I*_ex_, excitation intensity.

Light-driven polymerizations have enabled transformative advances in materials chemistry from coatings, adhesives, and lithography used in the fabrication of modern microelectronics to emergent additive manufacturing technologies (e.g., 3D printing) used broadly in the dental and medical industries to create items ranging from teeth alignment devices to surgical tools.^1−3^ Vat-based photopolymerizations, wherein rapid (∼seconds) solidification of liquid resin occurs upon exposure to light (i.e., photocuring), have led to some of the highest build rates of any 3D printing technique. Moreover, the precise spatial and temporal control offered by light has resulted in unparalleled print fidelity (i.e., feature resolution).^4−9^ Despite these impressive achievements, several challenges in light-based 3D printing persist. This includes a reliance on high-energy photons (<400 nm, UV) and/or high light exposure intensity (>100 mW/cm^2^), the need for inert printing conditions (e.g., N_2_ or Ar atmosphere), and the high cost of printing components (e.g., pulsed lasers) (Figure 1). These issues limit both accessibility, due to high cost, and materials scope, due to photodegradation, phototoxicity,^10^ and shallow penetration depth that arises from nonspecific absorption and scattering of short-wavelength UV light.^8,11^

In recent years, various strategies have been developed to address these particular limitations of UV photocuring. This includes photocatalysts that directly operate using visible and near-infrared light^12−17^ as well as more sophisticated multiphoton excitation modes for catalysts that require higher energy, such as two-photon absorption,^18^ two-step absorption,^19^ and photon upconversion.^20,21^ Notably, the latter three techniques have an inherent quadratic relationship between incident light intensity and photocuring rate. This superlinear relationship stems from the requisite combination of multiple photons to generate the radical species that initiate polymerization. In turn, photocuring can be accomplished within a focal point of light, which has allowed production of subdiffraction-limited features (roughly λ/2) and voxel-by-voxel (i.e., volumetric) 3D printing.^22^ However, to date, 3D printing in this manner has required scanning a high-intensity, tightly focused laser beam (>1 W/cm^2^), which is expensive (∼$10k–100k instrumentation) and results in slow fabrication (<10^–3^ mm^3^/s) of objects confined to small volumes.^22^

As an alternative 3D printing strategy to multiphoton laser-based methods, digital light processing (DLP) offers a low-cost route (∼$1k instrumentation) to the rapid fabrication (∼1 mm^3^/s) of large-volume objects.^22^ DLP 3D printing operates in a layer-by-layer fashion, where each exposure represents a 2D projection (i.e., slice) of the 3D object (Figure 1).^23^ Typical projection intensities in commercial DLP 3D printers range from ∼5 to 50 mW/cm^2^. However, higher-intensity light engines (>100 mW/cm^2^) represent an active area of research given the potential for higher production speeds that offset the additional energetic costs. Although the feature resolution of DLP 3D printing at present does not match that of multiphoton techniques, it can provide fidelity on the ∼10–100 μm scale with proper optimization. Specifically, feature optimization is accomplished by mitigating lateral (*x*, *y*) and vertical (*z*) overcure (i.e., solidification that occurs outside the defined irradiation zones). However, speed and resolution are often at odds within one-photon systems given that high light sensitivity is required to achieve rapid photocuring. Thus, scattered light and vertically transmitted light beyond a single layer often result in overcure, reducing feature fidelity.

Relative to standard DLP 3D printing, a multiphoton method has the potential to improve resolution, as the scattered and through-slice transmitted light lacks the intensity to induce curing given the superlinear dependence of the polymerization rate on the excitation intensity. Of the aforementioned multiphoton excitation pathways, triplet–triplet annihilation upconversion (TTA-UC) stands out due to its capability to operate efficiently (>10% quantum yield) under DLP-relevant low-intensity excitation (<50 mW/cm^2^).^24^ Indeed, TTA-UC-driven polymerizations have garnered considerable attention in recent years,^25−32^ but they have yet to be applied to DLP 3D printing. Instead, TTA-UC photocuring has recently been used for volumetric 3D printing. For example, Congreve and co-workers confined a TTA-UC system into silica-coated nanocapsules that were dispersed in a resin to print centimeter-scale objects,^33^ while Hayward and co-workers directly dissolved TTA-UC dyes into a resin for microscale fabrication using a focused LED source.^34^ However, despite these demonstrations, TTA-UC 3D printing has remained limited to high-intensity excitation (>10 W/cm^2^) and stringent oxygen-free conditions.^33−36^

Herein, our objective was to make upconversion-based 3D-printing more accessible by constructing a TTA-UC-driven photopolymerization system that achieves the desired superlinear (∼quadratic) intensity dependence (and hence high spatial resolution) upon exposure to low-intensity (<10 mW/cm^2^) visible light from an inexpensive LED source under ambient conditions. To acomplish this objective, we have developed a TTA-UC to Type I photoinitiation system through systematic resin formulation optimization and characterization. We also provide direct comparisons of the optimized TTA-UC resin to state-of-the-art Type I and Type II photoinitiation systems. Following optimization, the TTA-UC to Type I photosystem provides short gelation times (<60 s) with low-intensity green light exposure in the presence of ambient oxygen, which is competitive with Type II photosystems. With this resin formulation, we demonstrate for the first time the utility of TTA-UC in DLP-based 3D printing using a green LED to rapidly produce high-resolution objects (∼100 μm scale features). This showcases the potential for TTA-UC systems to provide a commercially viable platform for inexpensive light-based manufacturing.

## Results and Discussion

**Figure 2:**
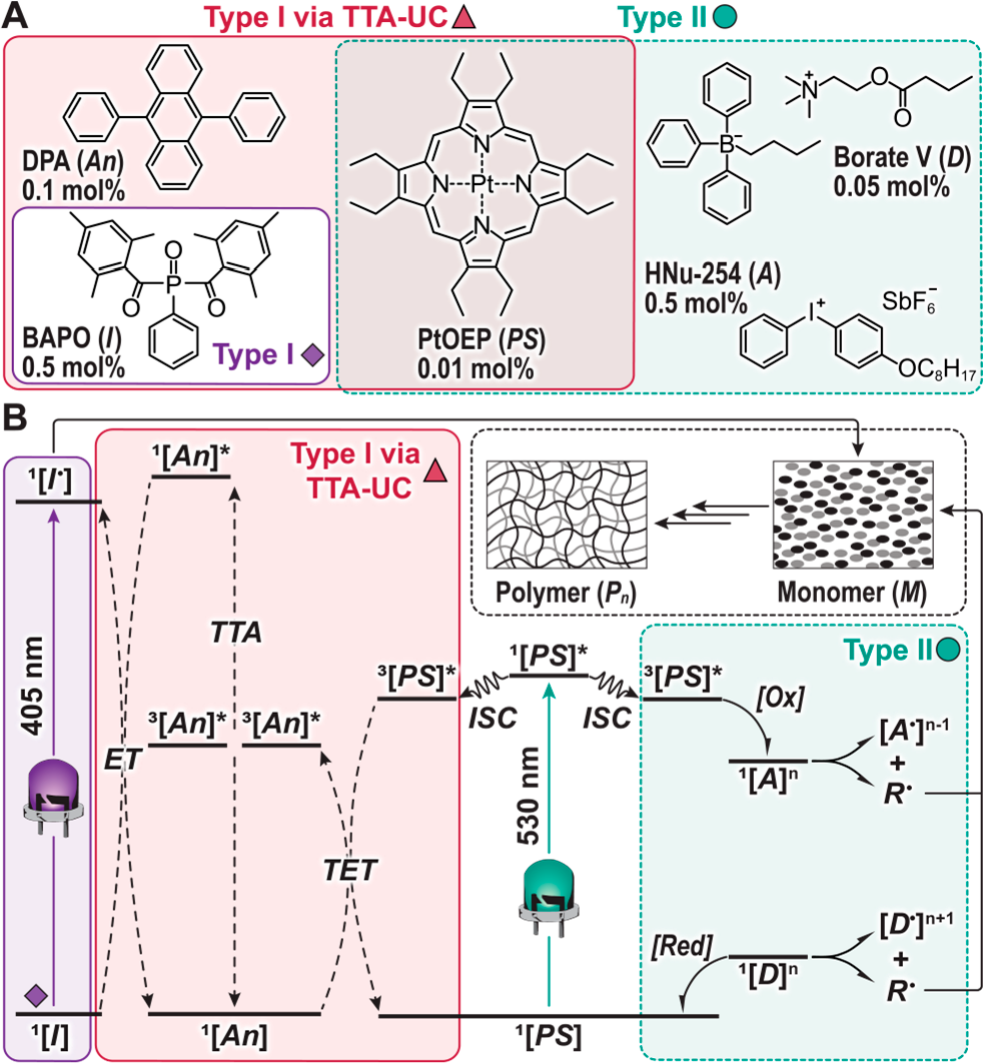
Photosystem components and photoinitiation mechanisms. (A) Chemical structures for Type I (left, violet), TTA-UC (center, red), and Type II (right, green) photosystems used in this study. **PS**, photosensitizer, platinum octaethylporphyrin (PtOEP); **An**, annihilator, diphenylanthracene (DPA); **I**, initiator, bisacylphosphine oxide (BAPO); **A**, electron acceptor, 4-octyloxydiphenyliodonium hexafluoroantimonate; **D**, electron donor, butyrylcholine butyltriphenylborate. (B) Energy level schematic showing the mechanisms for photoinitiation. Processes include photoexcitation, intersystem crossing (ISC), electron transfer (solid hook arrows), and energy transfer (dashed arrows). TET, triplet energy transfer; TTA, triplet–triplet annihilation; [Ox], oxidation; [Red], reduction; R^•^, radical; M, monomer; P_n_, polymer.

The photopolymerization system designed here couples two distinct processes, TTA-UC and Type I photoinitiation (i.e., unimolecular bond homolysis postexcitation) (Figure 2, red boxes). The TTA-UC cascade begins with absorption of a low-energy (∼525 nm, green light) photon by the photosensitizer (**PS**), platinum octaethylporphyrin (PtOEP). The resultant spin-singlet excited state (^1^[**PS**]*) of PtOEP then undergoes rapid intersystem crossing (ISC) to a long-lived spin-triplet excited state (^3^[**PS**]*). Next, this photoexcitation is transferred to 9,10-diphenylanthracene (DPA), a common annihilator (**An**) whose lower triplet energy relative to PtOEP (*E*_T,PtOEP_ = 1.91 eV, Figure S6; *E*_T,DPA_ = 1.77 eV)^37^ enables triplet energy transfer (TET) between these compounds. Triplet–triplet annihilation (TTA) can then proceed when two annihilators in their triplet excited state, ^3^[**An**]*, collide, yielding one ground-state annihilator, ^1^[**An**], and one annihilator in its singlet excited state, ^1^[**An**]*. The excited spin-singlet annihilator can then emit a high-energy photon (∼420–440 nm, violet-blue light, Figure S7) that can be absorbed by phenylbis(2,4,6-trimethylbenzoyl)phosphine oxide (BAPO) (Figure S8), an efficient Type I photoinitiator that does not absorb the low-energy green photons emitted by the LED (Figure S9) being used to excite PtOEP. In addition to emission reabsorption, BAPO can be activated via energy transfer from ^1^[**An**]*. In either case, the excited initiator (^1^[**I**]*) will cleave in a homolytic fashion to yield two radicals capable of initiating polymerization.

As a benchmark, this process was directly compared with an analogous Type II photopolymerization system driven by green light (Figure 2, green boxes). In this photosystem, PtOEP serves as a photoredox catalyst (labeled for simplicity as **PS**), while diphenyliodonium and *n*-butyl triphenylborate salts serve, respectively, as electron acceptor (**A**) and donor (**D**) components, completing an efficient redox cycle where radicals are generated on both ends. In addition, polymerizations driven via direct photoexcitation (∼405 nm, UV/violet light, Figure S9) of the Type I photoinitiator, BAPO, served as controls (Figure 2, violet boxes).

Several acrylate monomers were initially screened with the TTA-UC system, namely, isobornyl acrylate, dimethyl acrylamide, carbitol acrylate, and 2-phenoxyethyl acrylate (PhOEA). Of these four monomers, 2-phenoxyethyl acrylate provided the best combination of solubility and polymerization rate (Figure S10 and Table S1) and was therefore used for all further photophysical and polymerization studies. Notably, although uncommon in photocurable resins, 2-phenoxyethyl acrylate is an inexpensive monomer (as low as ∼$50/kg) derived from the commodity chemical phenoxyethanol,^38^ making it of viable utility in commercial additive manufacturing.

**Figure 3:**
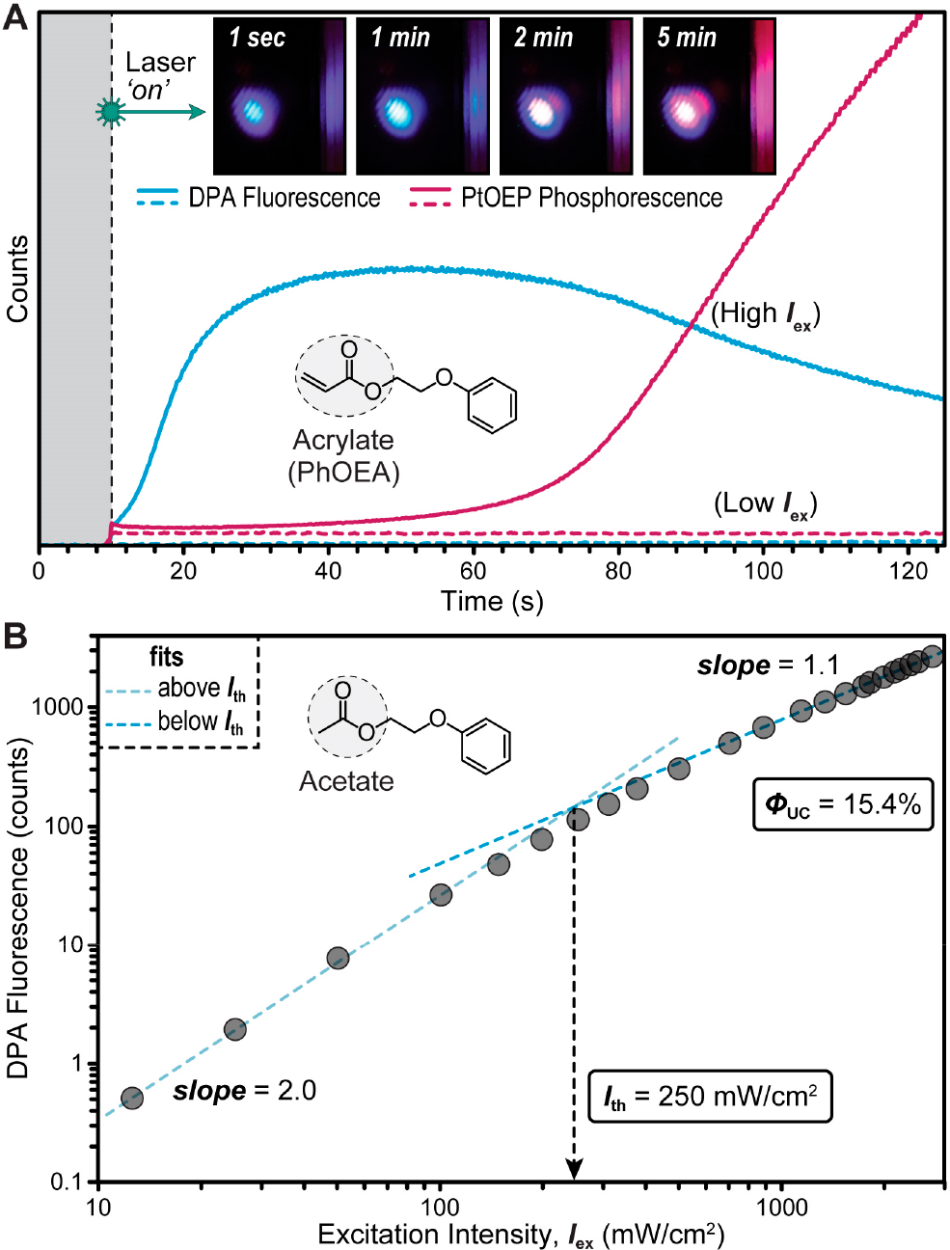
Characterization of PtOEP/DPA TTA-UC under an inert atmosphere using photoluminescence spectroscopy upon green (532 nm) laser excitation. (A) Time evolution of upconverted DPA fluorescence (cyan) and PtOEP phosphorescence (maroon) in 2-phenoxyethyl acrylate monomer. Photoluminescence was measured at 436 and 645 nm at low and high excitation intensity (*I*_ex_ = 50 and 1000 mW/cm^2^). (B) Upconverted DPA fluorescence in 2-phenoxyethyl acetate as a function of excitation intensity to quantify the threshold intensity (*I*_th_) using a double-logarithmic plot.

As a first step toward TTA-UC-based photopolymerization, we characterized the upconversion quantum efficiency (Φ_UC_) and intensity threshold (*I*_th_) of the PtOEP/DPA TTA-UC system via photoluminescence spectroscopy under an inert nitrogen atmosphere. To preclude DPA fluorescence quenching, measurements were performed in the absence of BAPO (i.e., using only PtOEP and DPA). To match the polymerization conditions described later, PtOEP and DPA were dissolved in 2-phenoxyethyl acrylate at concentrations of 0.6 and 6 mM, respectively. Exposure of this mixture to 50 mW/cm^2^ 532 nm green laser light resulted in no observable DPA fluorescence (Figure 3A), despite prior reports of TTA-UC occurring for PtOEP/DPA mixtures at <50 mW/cm^2^, albeit in more traditional solvents (e.g., toluene).^39^ Increasing the excitation intensity (*I*_ex_) to 1000 mW/cm^2^ resulted in DPA fluorescence monitored at 436 nm that grew in intensity during the first ∼30 s of exposure. After ∼60 s the DPA fluorescence decreased, concomitant with the appearance of a red emission band centered at ∼645 nm that was attributed to PtOEP phosphorescence. Upon concluding the experiment, the formation of solid inside the cuvette at the position of irradiation was observed, indicating that the PtOEP/DPA TTA-UC system can initiate photopolymerization in the absence of BAPO, although it requires high *I*_ex_ and exposure times (Figure S11). In the case of DPA, the energy of ^1^[**An**]* is too low to initiate acrylate polymerization, which was confirmed experimentally through direct excitation of DPA in monomer (Figure S12). As such, an alternate initiation pathway within the TTA-UC photosystem must exist, which we speculate could occur via electron transfer to monomer from a high-energy coupled triplet pair state that forms upon TTA^40−42^ prior to ^1^[**An**]*.

The time evolution of emission from the TTA-UC system (Figure 3A) was attributed to photoinduced electron transfer from DPA to acrylate, which was recently shown to be a viable initiation mechanism for photopolymerization.^26^ We hypothesize that upon light exposure, upconverted fluorescence quenching by 2-phenoxyethyl acrylate occurs, leading to the weak TTA-UC observed. Over time, the local concentration of monomer, acting as a DPA quencher, decreases due to polymerization, leading to a slow gain in DPA fluorescence with continued light exposure. Eventually, however, polymerization increases the viscosity of the solution to a point that diffusion-based TET from PtOEP to DPA and TTA between triplet excited DPA (^3^[**An**]*) molecules is severely hindered, leading to a decrease in DPA fluorescence and increase in PtOEP phosphorescence. Notably, the changes in photoluminescence from blue to red emission are visible to the naked eye, providing visual cues that uniquely enable qualitative tracking of this process (Figure S13 and Video S1).

Although the time-dependent emission behavior observed in 2-phenoxyethyl acrylate was unique, DPA quenching by the monomer precluded us from quantitatively determining Φ_UC_ and *I*_th_ for the PtOEP/DPA TTA-UC system. To bypass this issue, 2-phenoxyethyl acrylate was replaced with an analogous nonpolymerizable solvent, 2-phenoxyethyl acetate. Using the same concentrations of PtOEP (0.6 mM) and DPA (6 mM) as for photopolymerization (described below), the DPA fluorescence intensity at 436 nm was tracked as a function of 532 nm *I*_ex_ (Figure 3B). Plotting the results on a log–log scale revealed a change in slope from ∼2 to 1 at *I*_ex_ ≈ 250 mW/cm^2^, which identifies *I*_th_. Below *I*_th_, spontaneous decay of ^3^[**An**]* is rate-limiting, whereas above *I*_th_ TTA becomes the dominant and thus rate-limiting pathway.^24^ This behavior for TTA-UC systems can be described using eq 1:^43^
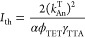
1where *k*_An_^T^ is the ^3^[**An**]* decay rate constant, α is the PS absorption coefficient, ϕ_TET_ is the TET efficiency from ^3^[**PS**]* to ^1^[**An**], and γ_TTA_ is the TTA second-order rate constant.

To estimate *I*_th_ in acrylate relative to the acetate (proxy) solvent, each component of eq 1 was systematically characterized apart from γ_TTA_, which was qualitatively compared between the two solvents based on the ^3^[**An**]* decay rates. Under equimolar conditions that provide high optical densities, α is similar in both acrylate and acetate. To characterize ϕ_TET_ in each solvent, time-resolved photoluminescence spectroscopy under an inert nitrogen atmosphere was used to measure changes in the PtOEP phosphorescence lifetime due to quenching by DPA (Figures S14–S17 and Table S2). In the absence of DPA, the lifetime of PtOEP was found to be ∼79 and ∼52 μs in acrylate and acetate solvents, respectively. The addition of 6 mM DPA to each solution decreases these values to ∼1.9 μs in both solvents, indicating that ϕ_TET_ is >96%. The *k*_An_^T^ values were extracted using transient absorption spectroscopy by monitoring the decay of an induced absorption signal from ^3^[**An**]* at 475 nm^44^ (Figures S18 and S19 and Table S2), which yielded lifetimes of 280 μs (*k*_An_^T^ ≈ 3570 s^–1^) and 122 μs (*k*_An_^T^ ≈ 8190 s^–1^) in acrylate and acetate, respectively. These lifetimes are considerably shorter than those obtained in common solvents with lower photosystem concentrations (e.g., ∼8 ms in toluene^45^). The shorter lifetimes observed in the present system are hypothesized to arise from DPA to PtOEP back energy transfer that is promoted by the high **PS** concentration we employ.^46^ This concentration was selected to match that used in the optimized resin (described below), which represents an application-oriented approach to designing TTA-UC systems.^21^ Furthermore, fitting the decay traces revealed that γ_TTA_ was larger in acrylate relative to acetate, indicating that a higher fraction of ^3^[**An**]* undergoes TTA in acrylate (Figures S18 and S19). Given the squared dependence of *I*_th_ on *k*_An_^T^ (eq 1), we estimate that *I*_th_ is <47 mW/cm^2^ in acrylate, which is >5× lower than acetate. Thus, the TTA-UC system was anticipated to operate effectively under excitation intensities relevant to DLP 3D printing. Further supporting this expectation was the high maximum Φ_UC_, which was measured as 15.4% in acetate, albeit at high *I*_ex_ values (3000 mW/cm^2^) (Figure S20). Notably, this Φ_UC_ value is out of a maximum of 50% (theoretical TTA-UC limit) and is conservative given a lack of correction for inner filter effects. Despite the stark disparity in *I*_th_ when using acetate versus acrylate as the solvent, we hypothesize that the maximum Φ_UC_ is comparable in these two solvents given the governing factors provided in eq 2:^47^
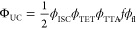
2where ϕ_ISC_ is the ISC efficiency of the **PS** (PtOEP), ϕ_TTA_ is the TTA quantum yield, *f* is the spin-statistical factor (i.e., the probability of forming an excited singlet state upon TTA^42^), and ϕ_fl_ is the **An** (DPA) fluorescence quantum yield. Given a common **PS** (PtOEP) that leverages spin–orbit coupling from the heavy Pt atom to drive ISC, ϕ_ISC_ was assumed to be similar in acrylate and acetate. As discussed previously, ϕ_TET_ is near unity in both systems due to the high **An** (DPA) concentration employed. Additionally, ϕ_TTA_ approaches unity at excitation intensities ≫ *I*_th_ irrespective of solvent.^45^ Finally, the effect of acrylate versus acetate solvent on ϕ_fl_ was probed by characterizing the fluorescence lifetime (τ_fl_) of DPA after 375 nm excitation (Figure S21). A τ_fl_ value of 6.8 ns was found in both solvents, which is nearly identical to τ_fl_ in toluene (6.9 ns), where ϕ_fl_ is near unity.^48^ This suggests that ϕ_fl_ should be similar and close to unity in both acetate and acrylate. Together, these results highlight that the present TTA-UC system operates efficiently with an uncorrected ϕ_UC_ of 15.4% in acetate (compared to ∼18–26% in other solvents),^49^ which, based on our spectroscopic results, is estimated to be of a comparable value in acrylate.

**Figure 4:**
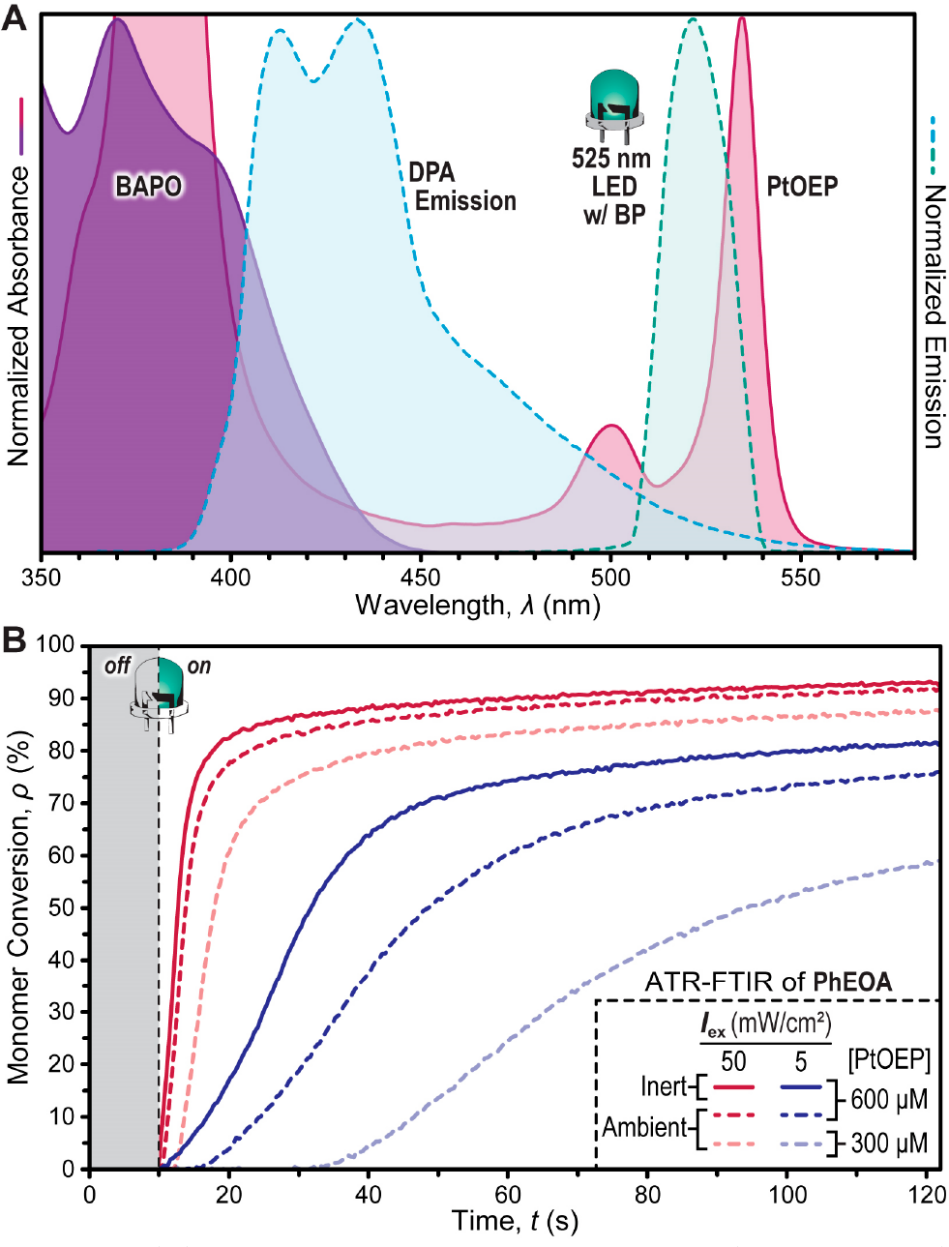
(A) The absorption spectrum of BAPO (purple-shaded) overlaps with the emission spectrum of DPA (cyan dashed). Likewise, the absorption spectrum of PtOEP (red-shaded) overlaps with the emission spectrum of the 525 nm LED used to excite the TTA-UC system. Note, the LED has been passed through a 525 × 25 nm bandpass filter to narrow its spectrum. (B) FTIR-ATR, plotting C=C conversion vs time of the standard resin ([PtOEP] = 600 μM; [DPA] = 6 mM; [BAPO] = 30 mM) and its 1/2 dilution. Resins were tested under inert (solid) and ambient (dashed) conditions and high intensity (50 mW/cm^2^, red) or low intensity (5 mW/cm^2^, blue) 525 nm light irradiation.

Having characterized the upconversion system, it was next employed to drive a classic Type I photopolymerization, using BAPO as the initiator (**I**), as its absorption spectrum shows good overlap with the emission spectrum of DPA (Figure 4A). A standard green LED (λ_max_ = 525 nm) was selected as the excitation light source, as its emission profile overlapped well with the absorption profile of PtOEP used in both the TTA-UC and Type II photosystems without being absorbed by BAPO. Additionally, the LED was equipped with a 525 × 25 nm bandpass (BP) filter to ensure selective absorption by PtOEP (Figure S9). However, it is worth noting that spectral overlap may not always be a direct predictor of photochemical reactivity,^50,51^ and thus, systematic controls (described below) were completed to confirm that the proposed TTA-UC to Type I initiation mechanism was indeed occurring.

Initial resin formulation optimization focused on maximizing the rate of polymerization (*r*_p_) by adjusting photosystem component concentrations. Real-time Fourier transform infrared spectroscopy (RT-FTIR) performed in an attenuated total reflectance (ATR) configuration was used to quantify *r*_p_ by measuring the conversion of monomer to polymer (ρ) via loss of the monomer C=C stretching band at ∼808 cm^–1^.^52^ Optimization via RT-FTIR kinetics led to a resin consisting of 0.01 mol % (∼600 μM) PtOEP, 0.1 mol % DPA, and 0.5 mol % BAPO, a **PS**:**An**:**I** ratio of 1:10:50 (Figures S22 and S23). The **PS** and **An** concentrations in the present system were similar to those used for upconversion in soft materials (∼500 μM and ∼13.4 mM, respectively),^53^ yet they are notably higher than what is traditionally employed for TTA-UC (∼5 μM and 1 mM, respectively).^45^ We rationalize the need for higher concentrations in our resins to overcome oxygen inhibition, operate in a viscous medium, and induce rapid curing upon exposure to low-intensity light, as required for 3D printing. Additionally, the **I** concentration corresponds to 1 wt % in the resin formulation, which is within the standard range of Type I photoinitiators used in photocurable resins (∼0.5–5 wt %).^11,15^ This **I** concentration falling at the lower end of the commercial use range has the added potential benefit of improving resin stability to accidental ambient light exposure.

Photopolymerizations with the optimized formulation were conducted using *I*_ex_ values of 5 and 50 mW/cm^2^ under both ambient and inert (argon) atmospheric conditions. In the absence of oxygen, excellent temporal control was observed, where polymerization began right after turning the LED “on” (Figure 4B). The *r*_p_, measured as the initial slope of monomer C=C conversion, was 109 ± 6.4 and 901 ± 41 mM/s for *I*_ex_ values of 5 and 50 mW/cm^2^, respectively (Figure 4B and Table S3), under inert conditions. As a control, resins without **An** (i.e., only **PS** and **I** present) did not polymerize upon exposure to the 525 nm LED (50 mW/cm^2^) (Figure S24). In contrast, under the same excitation conditions (525 nm LED, 50 mW/cm^2^), control resins without **I** (i.e., only **PS** and **An** present) showed a very slow polymerization (*r*_p_ = 5.3 ± 0.5 mM/s) (Figure S25), consistent with the aforementioned solid formation observed during Φ_UC_ characterization (Figure S11). Also of note, a red-shifted Type I acylgermane initiator (Ivocerin) was examined to increase the absorption overlap with upconverted DPA fluorescence (Figure S8). However, only modest improvements in *r*_p_ (≤15% increase) were measured relative to analogous resins with BAPO (Figure S26 and Table S4). Returning to the TTA-UC photosystem containing BAPO, the maximum C=C conversion (ρ_max_) was 82 ± 2% and 93 ± 1% for *I*_ex_ = 5 and 50 mW/cm^2^, respectively (Figure 4B and Table S3). The incomplete C=C conversion was attributed to the increase in viscosity upon polymerization, which hinders diffusion-limited processes. Notably, the 11% larger ρ_max_ observed when using the higher *I*_ex_ may be rationalized by the larger *r*_p_ and concomitant heat released from the exothermic reaction propagation, which can facilitate diffusion and thereby higher conversion.

Irradiating the same mixtures under ambient conditions resulted in *r*_p_ = 85 ± 7 mM/s and ρ_max_ = 75 ± 1% for *I*_ex_ = 5 mW/cm^2^, while increasing *I*_ex_ to 50 mW/cm^2^ boosted these values to *r*_p_ = 834 ± 34 mM/s and ρ_max_ = 92 ± 1% (Figure 4B and Table S3). Notably, the *r*_p_ at 50 mW/cm^2^ under ambient conditions was only 7% smaller than under inert conditions. This apparent low sensitivity to oxygen is attributed to triplet quenching rates that outcompete the rate of oxygen diffusion facilitated by the high **PS** concentration (∼600 μM), despite the relatively low viscosity (η) of the photopolymerizable resin (η_monomer_ ≈ 11 mPa·s), which was expected to enhance oxygen quenching. Moreover, DPA has been shown to act as a singlet oxygen scavenger.^54,55^ To the best of our knowledge, the low oxygen sensitivity is unprecedented for TTA-UC and provides a key avenue toward commercial additive manufacturing that is predominantly accomplished under ambient conditions. The effect of oxygen, however, is more apparent at *I*_ex_ = 5 mW/cm^2^, where an inhibition time (*t*_inh_) (i.e., period of no conversion after light “on”) of ∼4 s emerges (Figure 4B). The *t*_inh_ likely corresponds with the time it takes to fully consume oxygen in the irradiation zone. This effect was further pronounced upon diluting the photosystem to 1/2 of the original formulation (i.e., 300 μM PtOEP, Figure 4B) and 1/10 (i.e., 60 μM PtOEP, Figures S27–S30 and Table S5). At 1/2 the original concentration under ambient conditions, *r*_p_ was 56 ± 3 mM/s, ρ_max_ was 59 ± 2%, and *t*_inh_ was 21 ± 1 s for *I*_ex_ = 5 mW/cm^2^. Increasing *I*_ex_ to 50 mW/cm^2^ heightened these values to *r*_p_ = 504 ± 25 mM/s, ρ_max_ = 87 ± 1%, and *t*_inh_ = 2.0 ± 0.2 s under ambient conditions.

**Figure 5:**
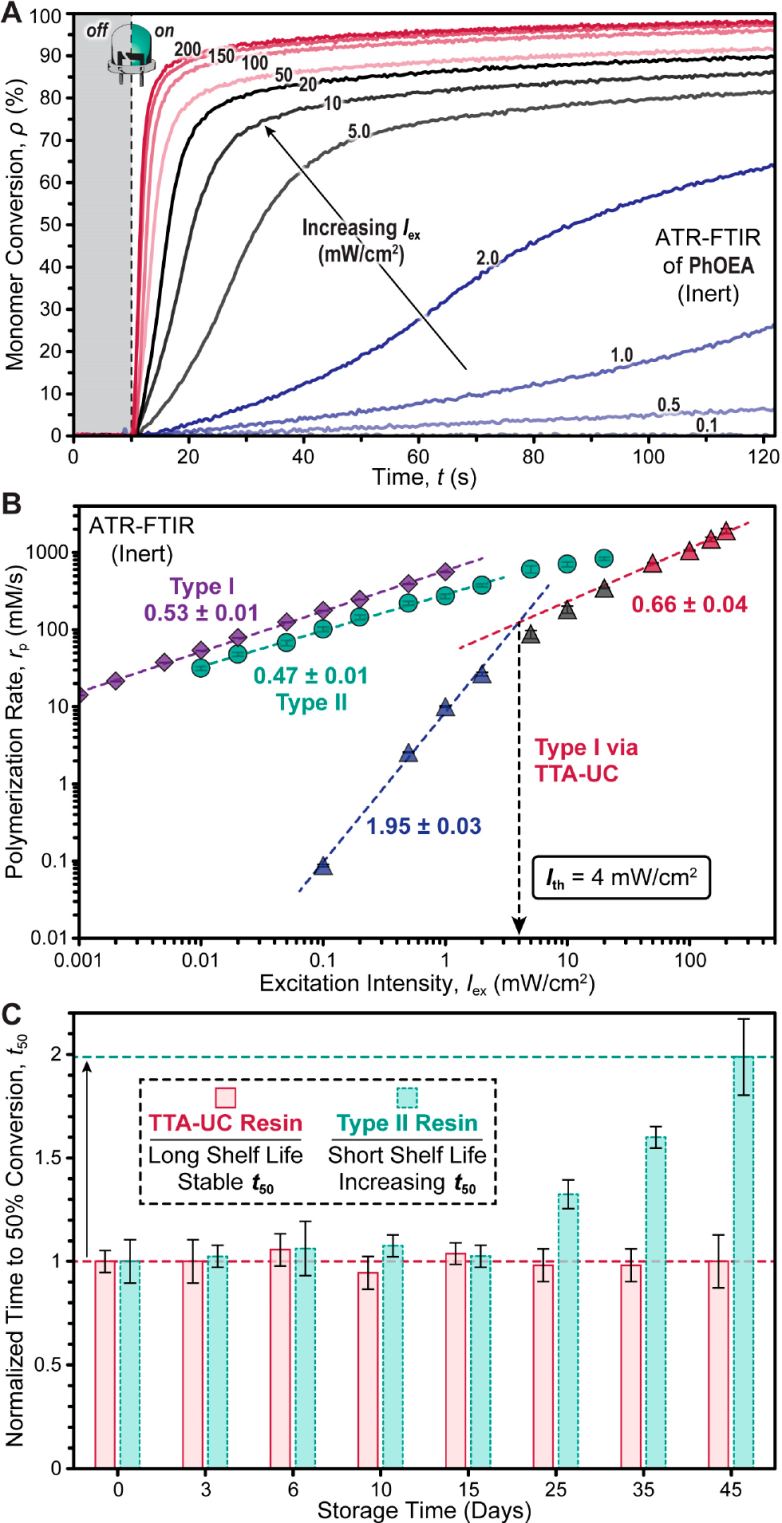
(A) FTIR-ATR intensity sweeps of the TTA-UC resin in 2-phenoxyethyl acrylate (PhOEA) under an argon atmosphere with the optimized photosystem ([PtOEP] = 0.6 mM; [DPA] = 6 mM; [BAPO] = 30 mM). The 525 nm LED was turned on at 10 s. (B) Plot of initial photopolymerization rate vs light intensity for the Type I photosystem driven by TTA-UC (triangles), Type I photosystem driven by direct UV/violet excitation of BAPO (diamonds), and the Type II photosystem (circles). The number near each linear best-fit curve indicates its slope. (C) Normalized time to reach 50% conversion (*t*_50_) of the Type II and TTA-UC resins over 45 days.

Given the large apparent influence of *I*_ex_ on the photopolymerization kinetics, we characterized its impact on the behavior of the optimized TTA-UC resin (Figure 5A). Samples were measured under an inert atmosphere to allow intrinsic TTA-UC characteristics, such as *I*_th_ and *r*_p_, to be quantified in the absence of oxygen inhibition. *I*_ex_ was varied by more than 3 orders of magnitude, from 0.1 to 200 mW/cm^2^. This resulted in *r*_p_ values ranging over 4 orders of magnitude, from 0.11 ± 0.01 to 2200 ± 150 mM/s. Plotting *r*_p_ versus *I*_ex_ on a double logarithmic scale revealed two linear regimes with distinct slopes that converge at *I*_ex_ ≈ 4 mW/cm^2^ (Figures 5B and S31–S33 and Table S6). In the low *I*_ex_ region (<4 mW/cm^2^), the slope is superlinear (1.95 ± 0.03), which lies between the expected slope of 1 and 3 for a two-photon-initiated polymerization. A slope of 1 would occur in an ideal two-photon process, while factors such as the presence of radical scavengers (e.g., oxygen) or radical occlusion (i.e., unimolecular termination^56^) can increase the slope up to 3.^34,57,58^ In the present system, trace oxygen is hypothesized to result in the measured slope >1 at low excitation intensities due to the inherent oxygen sensitivity of TTA-UC. In the high *I*_ex_ region (>4 mW/cm^2^), the slope becomes sublinear (0.66 ± 0.04), which is close to the expected slope of 0.5 for one-photon-initiated polymerizations that operate under conventional bimolecular termination events (i.e., *r*_p_ ∝ *I*_ex_^1/2^).^59,60^ The observable trends of *I*_ex_ on polymerization kinetics are analogous to that observed for upconverted photoluminescence via the TTA mechanism. Moreover, the two fitted slopes intersect at ∼4 mW/cm^2^ (≈*I*_th_), which is within the predicted range for *I*_th_ based on eq 1 but notably lower than the upper limit (<47 mW/cm^2^). Taken together, these results suggest that monitoring *r*_p_ as a function of *I*_ex_ provides an effective method to experimentally determine *I*_th_ for photopolymerizations driven by a two-photon process.

To confirm that the superlinear behavior was unique to TTA-UC-based Type I photopolymerizations, *r*_p_ values for the PtOEP/DPA/BAPO system were compared with those obtained by direct excitation of the benchmark Type I and Type II photosystems detailed in Figure 2 (Figure 5B). For the Type I photopolymerization, BAPO was employed as the photoinitiator at a concentration equimolar to that used in the optimized TTA-UC formulation (30 mM) and was photoexcited using a 405 nm UV/violet LED whose output was tuned to provide *I*_ex_ values from 0.001 to 1 mW/cm^2^. Likewise, Type II photopolymerizations were performed using a concentration of PtOEP equimolar to that employed in the optimized TTA-UC formulation (600 μM) along with 50 and 5 equiv (30 and 3 mM, respectively) of diphenyliodonium and *n*-butyl triphenylborate salts as the electron acceptor and donor, respectively. The same 525 nm LED with a 525 × 25 nm bandpass filter used for the TTA-UC resin was again employed for the Type II photosystem, with *I*_ex_ values ranging from 0.01 to 20 mW/cm^2^ to provide comparable *r*_p_ values.

Examining the performance of the Type I photosystem as a function of *I*_ex_ revealed *r*_p_ values from 17.3 ± 1.1 to 679 ± 2.0 mM/s and a sublinear relationship (slope = 0.53 ± 0.01) over the full range of *r*_p_ values when plotting on a double logarithmic scale (Figures 5B, S34, and S35 and Table S7). Similarly, the Type II system yields *r*_p_ values that display a sublinear dependence on *I*_ex_ (slope = 0.47 ± 0.01), which becomes even more pronounced (slope <0.3) at higher excitation intensities due to undesirable side reactions (Figures 5B and S36–S39 and Table S7). Although direct activation of both the Type I and Type II resins provided high *r*_p_’s at low *I*_ex_’s, the sublinear relationship between the two is expected to limit spatial resolution by facilitating overcure from scattered light and light transmitted beyond the depth of a single layer in the case of DLP (or other layer-by-layer) 3D printing. In contrast, the TTA-UC-based mechanism is anticipated to enhance spatial resolution given that scattered light and light transmitted through a layer can be tuned to fall below *I*_th_ where a superlinear relationship between *r*_p_ and *I*_ex_ exists.

Relevant to the translation of photocurable resins from lab to market is their stability. To this end, a TTA-UC resin with the optimal formulation was prepared, stored under ambient conditions (i.e., room temperature, atmospheric air, and in the dark), and tested using RT-FTIR periodically over the course of 45 days (Figures 5C and S40). Notably, little to no change in the photopolymerization kinetics and starting C=C absorption was observed, indicating excellent stability. In contrast, the analogous Type II resin containing PtOEP as the photoredox catalyst showed a steady increase in time to reach 50% conversion (*t*_50_) over the same time frame, ultimately resulting in a 2× increase in *t*_50_ after 45 days (Figures 5C and S41). Furthermore, no discoloration was observed for TTA-UC resins upon irradiation, while those from Type II resins became visually darker. This observation was consistent with photobleaching profiles measured using in situ UV–vis absorption spectroscopy, where PtOEP absorbance in Type II resins rapidly decreased upon green light exposure (Figures S42 and S43). This result indicates that **PS** stability is better for TTA-UC to Type I initiation relative to Type II photoredox mechanisms. This is attributed to energy transfer from ^3^[**PS**]*, obviating the formation of highly reactive **PS** radical ions that occur during electron transfer and present a likely avenue to degradation for the case of Type II photoredox-initiated polymerizations. These results showcase the stability of visible-light-reactive resins that undergo TTA-UC to Type I photoinitiation, which will enable their long-term storage and use in 3D printing applications.

**Figure 6:**
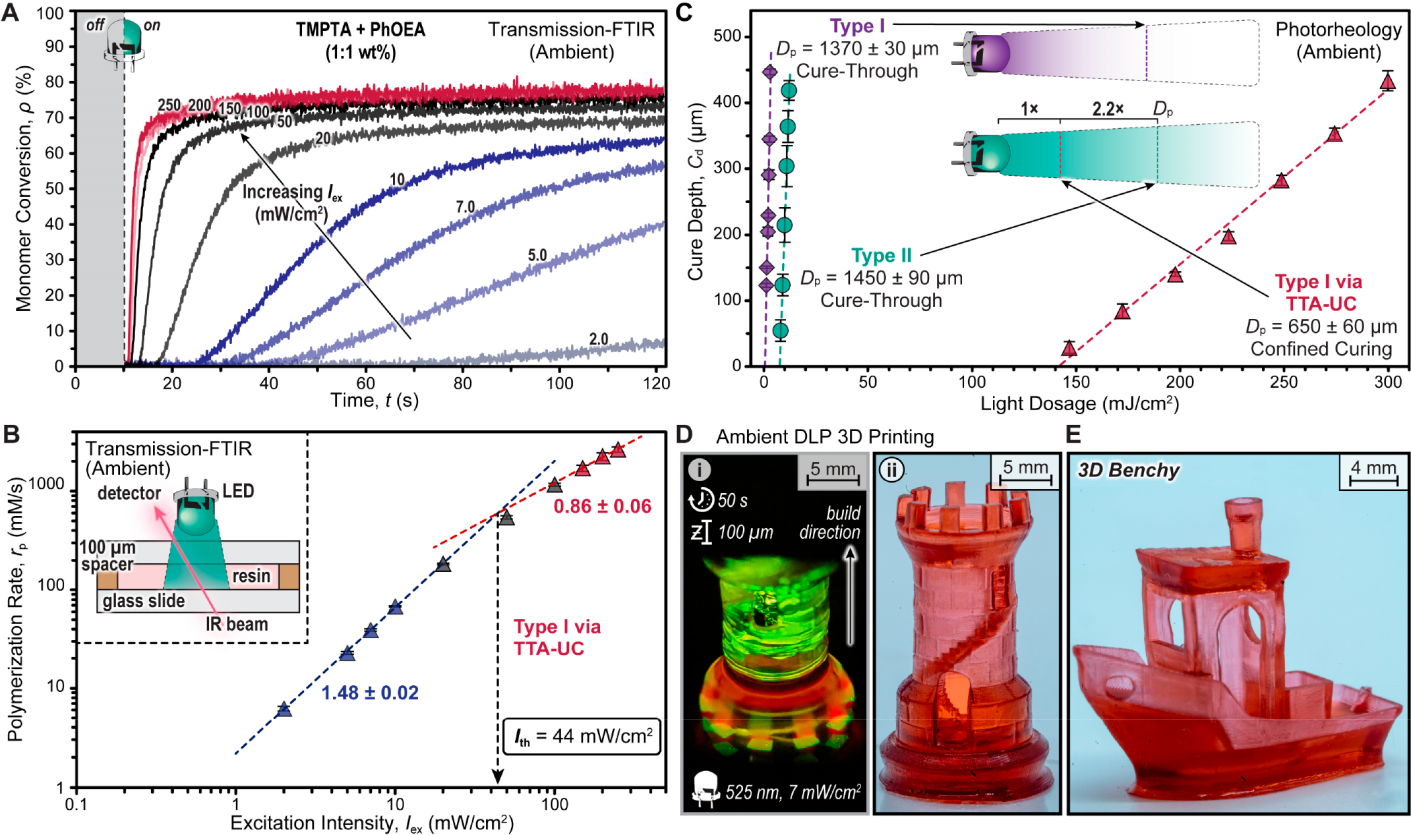
Testing of photocurable resins comprising trimethylolpropane triacrylate (TMPTA) and 2-phenoxyethyl acrylate (PhOEA) (1:1 w/w) under an ambient oxygen atmosphere with the optimized photosystem ([PtOEP] = 0.6 mM; [DPA] = 6 mM; [BAPO] = 30 mM). (A) FTIR-ATR intensity sweeps using the 525 nm LED turned on at 10 s. (B) Plot of initial photopolymerization rate (mM/s) vs light intensity (mW/cm^2^) for the Type I photosystem driven by TTA-UC. The number near each linear best fit curve indicates its slope. (C) Cure depth as a function of light dosage (*E*_0_) for the three resins. Critical exposure (*E*_c_) and depth of penetration (*D*_p_) values were determined from the semilog plot of *C*_d_ vs *E*_0_ in Figure S48. (D) Digital light processing (DLP) 3D printing under an ambient oxygen atmosphere of a Rook. (i) Active printing using a 525 nm LED, slice time of 50 s, and layer thickness of 100 μm followed by (ii) the completed print. (E) DLP 3D printed Benchy, using the same conditions as for the Rook.

To make the TTA-UC resin compatible with DLP 3D printing, we next incorporated trimethylolpropane triacrylate (TMPTA) as a cross-linker in a 1:1 weight ratio with 2-phenoxyethyl acrylate. Characterization of this resin was accomplished using transmission-mode RT-FTIR and photorheology for thin films (100 μm) under an ambient oxygen atmosphere to mimic layer curing in DLP 3D printing. Using the green LED with an *I*_ex_ of 10 mW/cm^2^, RT-FTIR of the cross-linkable resin formulation revealed an *r*_p_ of 100 ± 3.1 mM/s (Figure S44), which was within the same order of magnitude as the resin without cross-linker as measured using an ATR configuration, despite the presence of oxygen. Notably, polymerizations began shortly after turning the light on, with an average delay (*t*_inh_) of 4.0 ± 0.5 s. This short inhibition period was presumed to arise from triplet quenching by oxygen, and it could be further reduced upon increasing *I*_ex_ from 10 to 50 mW/cm^2^ (Figure S44). This was also supported by decreasing the concentration of BAPO, which led to lower polymerization rates but only a slight increase in *t*_inh_ (Figures S45 and S46). Furthermore, characterizing *r*_p_ for this system (100 μm thickness in ambient oxygen) at several different intensities revealed an *I*_th_ of ∼44 mW/cm^2^ (Figure 6A,B and Table S8), which was an order or magnitude larger than that measured using ATR under inert atmospheric conditions (*I*_th_ = 4 mW/cm^2^). Among the intensities measured, *I*_ex_ = 7 mW/cm^2^ matched that used for 3D printing, which gave *r*_p_ = 48 ± 1.3 mM/s and *t*_inh_ = 21 ± 0.3 s. Using these same conditions (*I*_ex_ = 7 mW/cm^2^, thickness = 100 μm), photorheology was used to estimate the minimum irradiation time required for curing (i.e., gel point), which was characterized by crossover of the storage (*G*′) and loss (*G*″) moduli. Under these conditions, the average gel point was 39.6 ± 2.4 s (Figure S47), which is within a reasonable time frame for DLP 3D printing.

Expanding upon these initial photocuring results, a series of systematic photorheology experiments were performed to understand the influence of the photopolymerization mechanism (TTA-UC, Type I, and Type II) on vertical (*z*) overcure (Figures 6C and S48 and Table S9). To this end, a series of cure depth (*C*_d_) experiments going beyond a single desired 100 μm layer were performed, holding constant (to the best of our ability) key factors influencing *C*_d_ outside the photosystem composition and associated mechanism. Specifically, *I*_ex_ from the 405 nm LED for Type I and 525 nm LED for Type II and TTA-UC was set to 0.1, 0.5, and 10 mW/cm^2^, respectively, to provide similar *r*_p_ values of ∼220 mM/s, as measured using RT-FTIR (Figure S49). Furthermore, the 405 and 525 nm light-absorbing components (i.e., BAPO and PtOEP, respectively) provided resins with corresponding average LED transmittance values for a 100 μm path length of 78% and 79%, respectively, across the full wavelength range of spectral overlap (i.e., considering chromophore absorbance and LED emission, see Figures S50–S52 and Optical Density Matching in the SI for more details). The *C*_d_ values were characterized as a function of light exposure time for each resin. After irradiation of the resin sitting in a 500 μm gap between static parallel plates, unreacted monomer was wicked away, and the upper plate was lowered stepwise (1 μm/s) until reaching a set force of 2 N,^61^ upon which the gap height was equated to *C*_d_ (Figure 6C). Next, intrinsic resin properties influencing *C*_d_ were determined using the Jacobs equation (eq 3):^62^
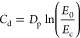
3where *D*_p_ corresponds to the penetration depth of light, *E*_c_ is the critical exposure energy required to cure an infinitesimal layer, and *E*_0_ is the experimental exposure energy (i.e., light dosage).^61,63,64^

Plotting *C*_d_ vs *E*_0_ for each of the three resin formulations revealed a stark disparity in the light dosage required to cure a given depth (=slope) and the critical exposure *E*_c_ (=*x* intercept) for TTA-UC relative to the Type I and Type II controls (Figures 6C and S48). Due to its lower quantum efficiency of initiation, TTA-UC-driven Type I has an *E*_c_ that is ∼20× higher than that of the Type I and II systems. Importantly, however, despite the higher energy requirement for TTA-UC, its associated *D*_p_ of 650 ± 60 μm is ∼2× lower than for Type I (1370 ± 30 μm) and Type II (1450 ± 90 μm). Given the nearly matched optical densities for the 525 and 405 nm absorbing resins, one would anticipate equivalent *D*_p_ values of ∼900 μm based on eq 4:
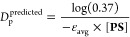
4where ε_avg_ is the average molar absorptivity based on the overlap between the green LED emission and **PS** or violet LED emission and **I** absorption profiles (see Optical Density Matching for more details). However, this equation was created to correlate *D*_p_ to *C*_d_ for traditional free-radical polymerizations, where a sublinear relationship between *I*_ex_ and photocuring exists. The considerably lower *D*_p_ for TTA-UC is hypothesized to arise from the superlinear relationship between *I*_ex_ and *r*_p_. In turn, this should correspond with reduced vertical overcure (i.e., cure-through) from light transmitted beyond a single print layer while also providing a mechanism to reduce lateral overcure from scattered light. This form of photocuring confinement is distinct from traditional absorptive strategies, where opaquing agents (i.e., dyes that act as passive absorbers) are added to 3D printing resins to attenuate light.^12−15^ As such, improved resolution for layer-by-layer 3D printing relative to conventional one-photon curing processes is anticipated when using the TTA-UC to Type I resin.

As a final proof of concept, the TTA-UC photosystem was employed for DLP 3D printing under conditions comparable to those used for the aforementioned characterization: a green LED having a λ_max_ of 525 nm, full width at half-maximum (fwhm) of 34 nm, and maximum *I*_ex_ of 7 mW/cm^2^ (Figure S53). Printing was performed under ambient conditions with no resin deoxygenation. An initial print file containing 12 discrete sections with preprogrammed light exposure times from 5 to 115 s per 100 μm layer was used to determine the optimal exposure time (Figure S54). From this, it was found that a minimum exposure time of 45 s/100 μm layer was required to fabricate objects with lateral (*x*, *y*) features as small as 100 μm, which corresponds to ∼4–5 pixels on the projected image. Notably, there was little observable lateral overcure at exposure times >45 s, which may arise from the two-photon nature of the photocuring process. Using an exposure time of 50 s/100 μm (∼7 mm/h), two complex prints containing several overhangs and small holes were targeted. Specifically, a Rook that contained an internal spiral staircase and a 3D Benchy with small (∼4 mm diameter) portholes on the hull and a hollow smokestack were printed (Figure 6D,E). For the 3D Benchy, the entire print (1.0 cm^3^) took 3 h and 10 min to complete (build rate = 0.32 cm^3^/h). This result is comparable to that previously reported for the same object produced by Congreve and co-workers (∼0.1 cm^3^, build rate = 0.05 cm^3^/h)^33^ while showing a qualitative improvement in feature resolution using the present method. Moreover, none of the TTA-UC to Type I prints required incorporation of photostable opaquing agents to achieve high-resolution features (i.e., mitigate overcure), which simplifies the resin formulation and paves an avenue to semitransparent and colorless objects with further optimization, such as lowering **PS** concentration and/or identifying bleachable **PS**s. These DLP 3D prints showcase the potential of TTA-UC to Type I photocuring as a new mechanism for rapid, low-intensity, and high-resolution manufacturing.

## Concluding Remarks

A Type I photocurable resin driven by TTA-UC with low intensity (∼10 mW/cm^2^) green light (∼525 nm) that operates under an ambient oxygen atmosphere was demonstrated for the first time, which enabled rapid, high-resolution DLP 3D printing. This advance was facilitated by the high upconversion quantum efficiency (∼15%) and low upconversion threshold (<50 mW/cm^2^) of the resin’s PtOEP/DPA components. Optimization of the resin formulation using RT-FTIR spectroscopy under an inert argon atmosphere revealed that a 1:10:50 PtOEP:DPA:BAPO ratio, where [PtOEP] = 600 μM, gave a rapid polymerization (∼100 mM/s) at low light intensity (∼10 mW/cm^2^). Importantly, the polymerization rate of the TTA-UC to Type I system exhibited a quadratic dependence on light intensity. This feature can enable improved spatial resolution in 3D printing over that achievable with analogous Type I and II photosystems by mitigating curing that occurs outside of the desired irradiation zone from low-intensity transmitted and scattered light. Further supporting translation to 3D printing was excellent shelf stability of the TTA-UC resin, with no change in photopolymerization performance observed during a 45-day study.

Our work additionally poses several exciting fundamental questions and prospective avenues to examine and leverage TTA-UC in photopolymerizations. For example, we observed polymerization in the absence of BAPO initiator and speculated that it was driven by electron transfer from high-energy triplet pair states to 2-phenoxyethyl acrylate monomers. As this represents a new pathway for upconversion-driven chemistry to operate beyond the spin-statistics constraints of traditional ^1^[**An**]* formation,^40,42,65^ it could provide a new paradigm in photopolymerizations that do not require traditional initiators. Additionally, alternative **PS**, **An**, and **I** pairs, along with oxygen scavenging additives,^14^ can be examined to eliminate the use of precious metals^66,67^ while increasing Φ_UC,_*r*_p_, and anti-Stokes shift to reduce energetic losses, widen the range of excitation wavelengths, increase photocuring efficiency, and improve 3D printing speed and resolution. As a result, we anticipate that TTA-UC to Type I photocuring can be harnessed to enable benign and wavelength-selective fabrication of multifunctional materials with potential applications in medicine, from tissue engineering for improved disease models to soft actuators for human-interfaced robotics and electronics.

## Supporting Information Available

Additional experimental (materials, methods, and instrumentation) and characterization details (PDF)
Spectral evolution of the TTA-UC photopolymerization without photoinitiator (MP4)
Side-by-side comparison of emission from TTA-UC printing resin during transmission FTIR (MP4)

## Abbreviations

LED: light-emitting diode
DLP: digital light processing
*I*_scatter_: scattered light intensity
*r*_p_: rate of polymerization
*I*_ex_: excitation intensity
TTA: triplet–triplet annihilation
UC: upconversion
**PS**: photosensitizer
PtOEP: platinum octaethylporphyrin
DPA: diphenylanthracene
**An**: annihilator
TET: triplet energy transfer
**I**: initiator
BAPO: bisacylphosphine oxide
**A**: electron acceptor
**D**: electron donor
PhOEA: 2-phenoxyethyl acrylate
ISC: intersystem crossing
Ox: oxidation
Red: reduction
M: monomer
P_n_: polymer
Φ_UC_: upconversion quantum efficiency
*I*_th_: intensity threshold
*k*_An_^T^: ^3^[**An**]* decay rate constant
α: PS absorption coefficient
Φ_TET_: ^3^[**PS**]* to ^1^[**An**] TET efficiency
γ_TTA_: TTA second-order rate constant. Φ_ISC_, ISC efficiency of **PS**
Φ_TTA_: TTA quantum yield
*f*: spin-statistical factor
ϕ_fl_: fluorescence quantum yield of **An**
BP: bandpass
RT: real-time
FTIR: Fourier transform infrared spectroscopy
ATR: attenuated total reflectance
ρ: monomer to polymer conversion
ρ_max_: maximum conversion
η: viscosity
*t*_inh_: inhibition time
*t*_50_: time to reach 50% conversion
TMPTA: trimethylolpropane triacrylate
*C*_d_: cure depth
*E*_c_: critical exposure
*D*_p_: depth of penetration
*E*_0_: total light energy
fwhm: full width at half-maximum
