# Supplementary material for: Triplet Upconversion under Ambient Conditions Enables Digital Light Processing 3D Printing
Source: ACS Cent Sci. 2024 Jan 16;10(2):272–82. doi: 10.1021/acscentsci.3c01263 (PMC10906251; doi:10.1021/acscentsci.3c01263)
Supplement: Supplementary file 1 — oc3c01263_si_001.pdf [file oc3c01263_si_001.pdf]

Supporting Information

Triplet Upconversion Under Ambient Conditions Enables Digital Light Processing 3D Printing

Connor O'Dea, Jussi Isokuortti[a], Emma E. Comer Sean T. Roberts*, Zachariah A. Page*
Department of Chemistry, The University of Texas at Austin, Austin, TX, 78712 USA
***Corresponding authors:** zpage@cm.utexas.edu, roberts@cm.utexas.edu

Table of Contents

EXPERIMENTAL DETAILS S2-S6

Materials S2

Instrumentation S2-S6

Light Emitting Diodes (LEDs) S6

CHARACTERIZATION S7-S36

Absorption & Emission S7-S8

RT-FTIR for Monomer Selection S9

Upconversion Quantum Yield S10-S11

Transient Absorption Spectroscopy S12-S15

Fluorescence Lifetime S16

ATR-FTIR Characterization (Inert) S17-S29

UV/Vis Photodegradation (Ambient) S30

Transmission FTIR Characterization (Ambient) S31-S32

Rheology Studies S33-S37

3D Printing S38

Video Captions S39

REFERENCES S40

EXPERIMENTAL DETAILS

Materials

Chemicals. All reagents were used as received unless otherwise noted. 2-Phenoxyethyl acrylate (stabilized with MEHQ) (>93%) was purchased from TCI. 2-Phenoxyethylacetate (98%), Platinum(II) 2,3,7,8,12,13,17,18-octaethyl-21H,23H-porphyrin (PtOEP, ≥97%), 9,10-Diphenylanthracene (97%) were purchased from Sigma-Aldrich. Trimethylolpropane triacrylate (TMPTA, ≥88%) was purchased from Alpha Aesar. Phenylbis(2,4,6-trimethylbenzoyl)phosphine oxide (BAPO, 99%) was purchased from AmBeed. Bis(4-methoxybenzoyl)diethylgermanium (ivocerin, >99%) was purchased from Synthon Chemicals. Borate V and H-Nu 254 were purchased from Spectra Group Limited.

Instrumentation

UV-Visible Absorption Spectrometer. Steady-state optical characterization was accomplished by collecting UV-visible absorption spectra on an Ocean Optics (QE PRO-ABS) Fiber Optic Spectrometer utilizing deuterium-tungsten halogen light sources (DH-2000-BAL). 600 μm fiber-optic cables (QP600-025-SR) were coupled to the detector with a slit width of 10 μm. Dilute absorption data was collected using quartz cuvettes (1 cm path length) and an Ocean Optics sample holder (qpod2e).

Emission Spectrometer. Fluorescence and phosphorescence spectra were recorded on a Horiba Fluorolog-3 spectrofluorometer. The optical upconversion measurements were performed with a custom-built setup that uses a Coherent Verdi V-10 532 nm laser as an excitation source and Ocean Optics Flame spectrometer as a detector (**Figure S1**). The emission was collimated and focused with two plano-convex lenses to a 600 μm fiber-optic cable (QP600-025-SR) connected to the spectrometer. The intensity of the laser excitation was modulated with a Newport variable neutral density filter and by changing the laser power. The excitation power was measured with a Thorlabs SV-120VC power sensor and PM100D console and the beam diameter was 2.25 mm according to the manufacturer's specification. Fluorescence lifetimes were recorded using a time-correlated single photon counting module of the Fluorolog-3 spectrofluorometer with a Horiba NanoLED pulsed diode laser (375 nm, 1 MHz pulse frequency).

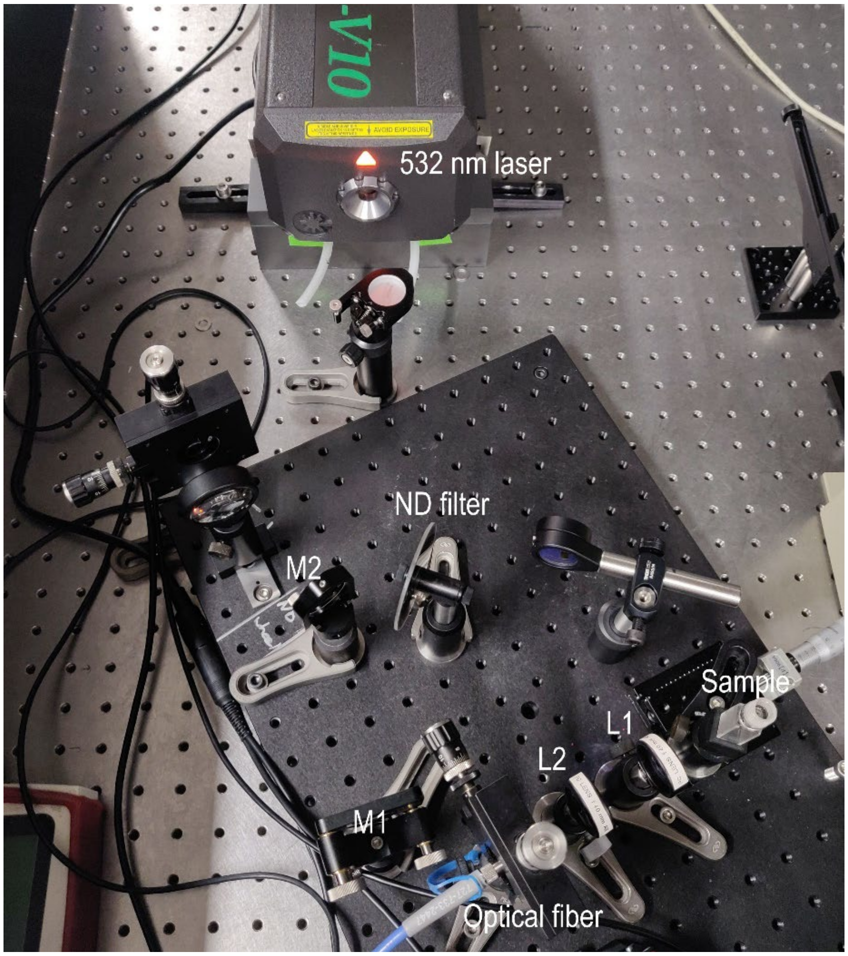

Figure S1. Upconversion setup used to measure the upconversion quantum yield and intensity threshold in 2-phenoxyethyl acetate. The laser beam is reflected with mirrors M1 and M2 through the variable neutral density (ND) filter to the sample cuvette. The emission is collected at a 45-degree angle with lenses L1 and L2 through an optical fiber to the spectrometer.

Transient Absorption Spectrometer. Triplet lifetimes were measured using a Magnitude Instruments enVISion transient absorption spectrometer using a 532 nm pulsed excitation laser (800-1000 Hz, 0.23-0.4 $\mu J/cm^2$ pulse fluences, measured with Thorlabs SV-120VC power sensor and PM100D console). The spectrometer can be used to measure both transient absorption and time-resolved photoluminescence.

Real-Time Fourier Transform Infrared (RT-FTIR) Spectrometer. RT-FTIR was recorded utilizing an INVENIO-R FT-IR Spectrometer from Bruker (**Figure S2**) and controlled via OPUS Spectroscopy Software. A liquid nitrogen cooled (LN-MCT Mid) detector was used for measurements. Surface FTIR characterization of solids and liquids under an argon atmosphere during illumination is accomplished using a GladiATR Illuminate accessory (SKU 026-1800) from PIKE Technologies (**Figure S2**) that couples to the Bruker INVENIO through Quick-Lock recognition. Samples are irradiated through the bottom of a diamond crystal using an LED transmitted through a 3 mm liquid light guide (LLG3-4Z) from ThorLabs. Illumination occurs from under a diamond attenuated total reflectance (ATR) element, which sits in a heated plate with a maximum temperature of 210 °C. The angle of incidence of the IR probe beam is 45°, the diamond crystal surface is 3 mm, and the spectral range is 4000 to 30 cm^{-1}. The sampling depth was calculated to be 2.6 μm using an on-line calculator from PIKE Technologies (https://www.piketech.com/pikecalc/). FTIR characterization of thin films (100 μm) was performed using a transmission accessory (A043-N/Q) from Bruker (**Figure S3**). Samples sit horizontally and are irradiated using an LED transmitted through a 5 mm liquid light guide (LLG5-4Z) from ThorLabs.

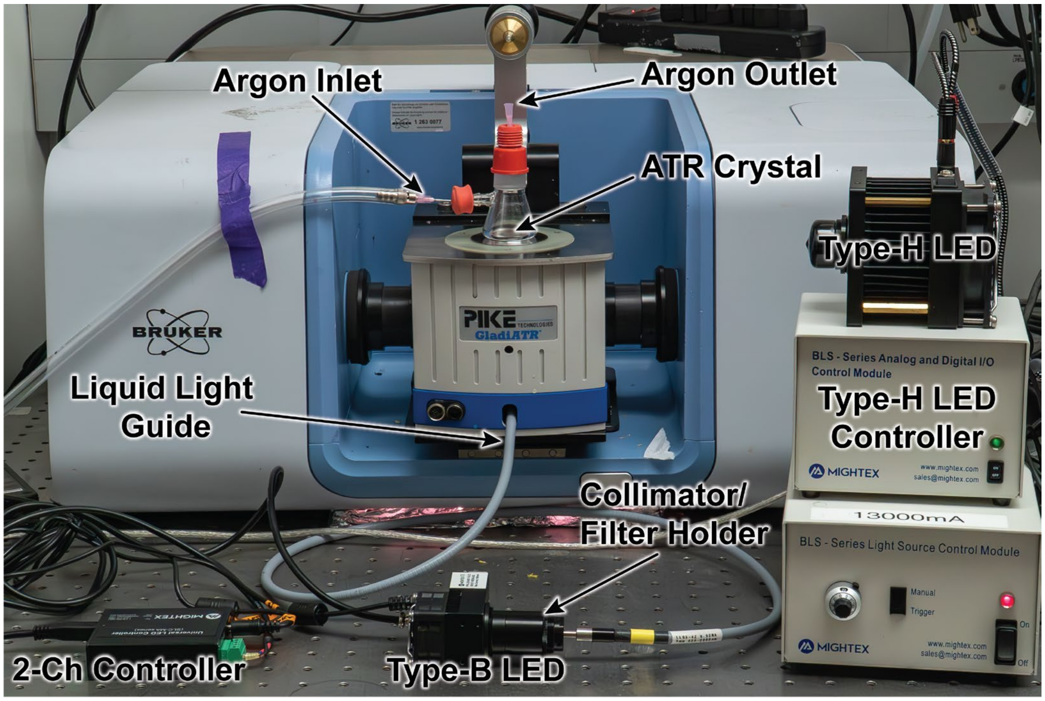

Figure S2. ATR-FTIR accessory allowing for LED irradiation at sample interface. Glass cover used for inert sample environment. Type-B LED (530 nm) used for intensities <50 mW/cm^2, Type-H LED (525 nm) used for intensities >50 mW/cm^2. A 525×25 nm bandpass filter used in both configurations.

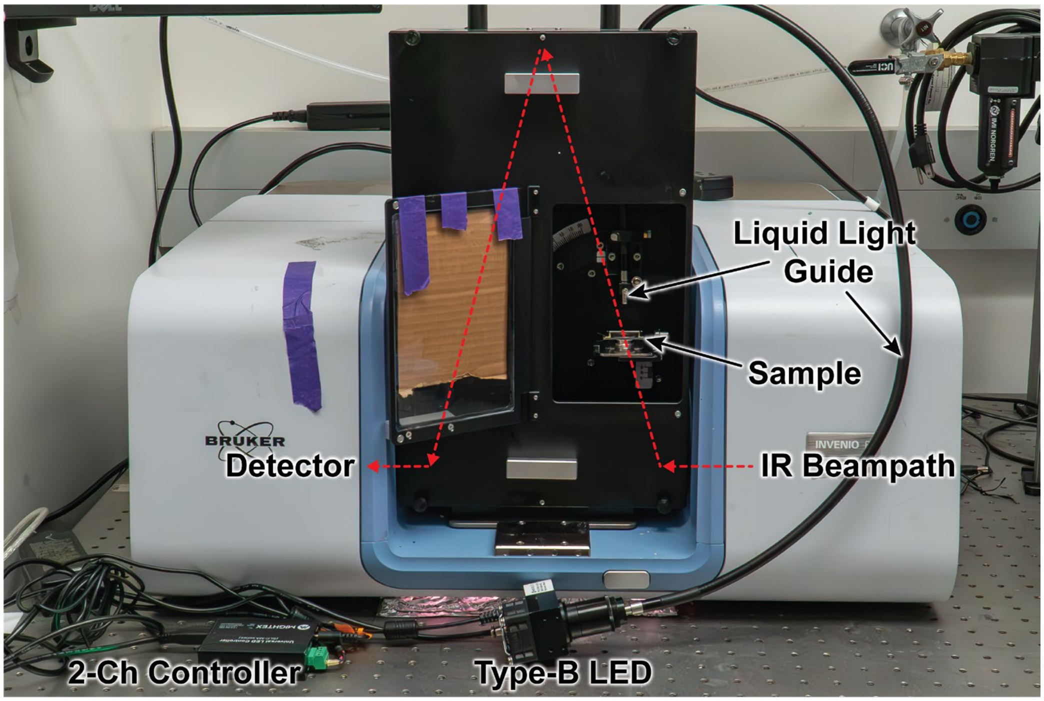

Figure S3. Bruker Transmission FTIR accessory allowing for top-down irradiation of a sample.

Rheometer. The Discovery Hybrid Rheometer (DHR) 20 from TA Instruments (serial # 5343-0845) equipped with a HR x0 Upper Peltier Plate System (part # 534050.901) and 25 mm stainless steel geometry

(part # 534516.941). The UV Light Guide Accessory (part # 546301.901) and acrylic bottom plate (part # 403064.901) were used for photorheology experiments (**Figure S4**). Calibration was done using Canon S60 (low-viscosity) and S600 (high-viscosity) oils as standards and data collected and analyzed using the TRIOS software. The minimum torque is 1 nN.m for oscillation and 3nN.m for steady sheer, both have a maximum of 200 mN.m with 0.1 nN.m resolution. The frequency range is 1.0×10^{-7} – 100 Hz. The angular velocity range is 0 – 300 rad/s. The maximum normal force is 50 N with a sensitivity of 0.005 N and resolution of 0.5 mN.

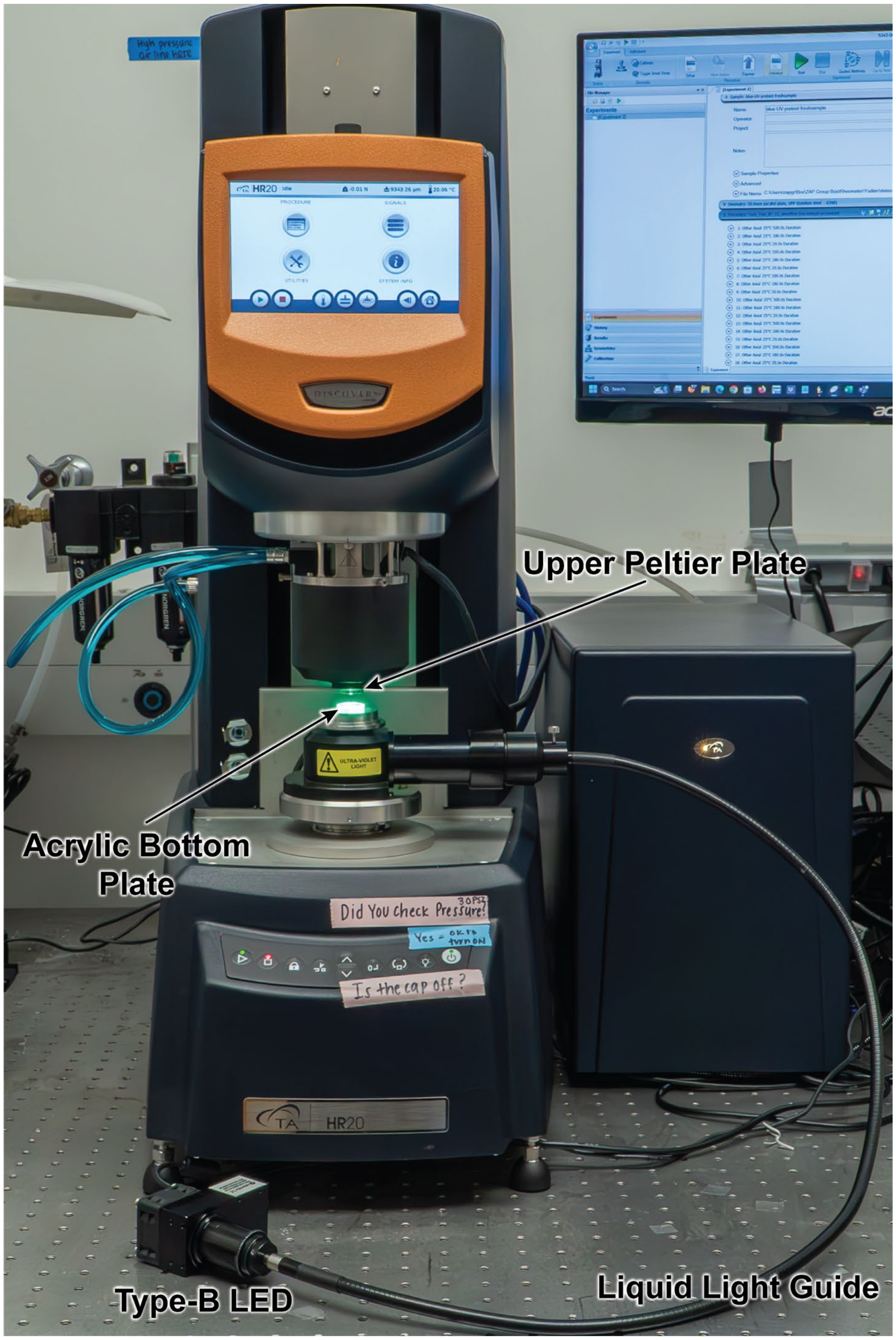

Figure S4. Rheometer with light guide accessory used for gel point and cure depth experiments.

DLP 3D Printing. 3D printing was performed using a custom-made, DLP-based 3D printer (Monoprinter, MA, USA) (**Figure S5**). Detailed information about the 3D printer can be found in our previous report.[S1] The Printer was equipped with an exchangeable visible LED centered at 525 nm (green, Luminus PT-120-G) with a full width at half maximum (FWHM) of 34 nm (**Figure S51**). The projector resolution was 1920 × 1080 pixels, with each pixel being 20 μm × 20 μm at the image plane. The minimum achievable layer thickness is 25 μm. Here, prints were primarily performed using a layer thickness of 100 μm. The DLP employed here follows a layer-by-layer additive manufacturing process, where each 100 μm layer is cured between an upper cured layer or build platform and a transparent fluorinated polymer film (Teflon FEP film, DuPont, 127 μm thick) as the base of the resin tank that provides a non-stick and somewhat flexible surface for detachment.

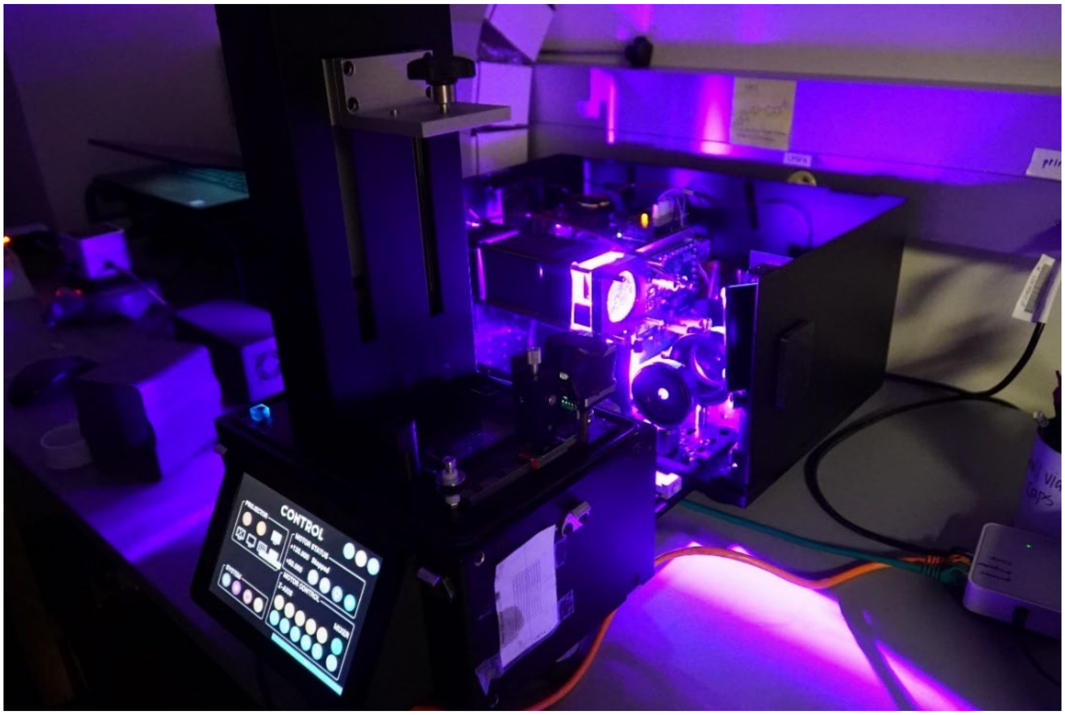

Figure S5. Custom 1-Channel DLP 3D printer with interchangeable LEDs.

Light Emitting Diodes (LEDs)

All LEDs used were purchased from Mightex Systems. The product numbers for the three LEDs are LCS-0405-12-22 (405 nm), LCS-0530-15-22 (530 nm), and LCS-0525-60-22 (525 nm) and powered by an LED Controller (SLC-MA02-U). Green LEDs were equipped with a 525×25 nm bandpass filter from Edmund Optics (#87-789). Lightguide Adapters from ThorLabs were used to irradiate resins via either a 3 mm liquid light guide (LLG-3-4Z) when using the ATR accessory or a 5 mm liquid light guide (LLG5-4Z) when using the transmission accessory. The emission profile for each LED was measured using a calibrated UV-Vis Ocean Insight system. Irradiation intensities were measured with a Thorlabs PM100D photometer equipped with silicon-based photodiode power sensor (S130C, Thorlabs) prior to each experiment.

CHARACTERIZATION

Absorption & Emission

The absorption spectra of PtOEP (**Figure S6**), DPA (**Figure S7**), BAPO, and Ivocerin® (**Figure S8**) were measured using a 1 cm cuvette. Samples used to record emission spectra of PtOEP (**Figure S6**) and DPA (**Figure S7**) were prepared in a 1 cm cuvette at a concentration that gave a maximum absorbance of 0.1 in the excitation and emission range to exclude inner filter effects. The PtOEP sample was bubbled with nitrogen for 20 minutes prior to measuring. Emission spectra of the 405 nm and 525 nm LEDs measured with the Ocean Optics (QE PRO-ABS) Fiber Optic Spectrometer are shown in **Figure S9**.

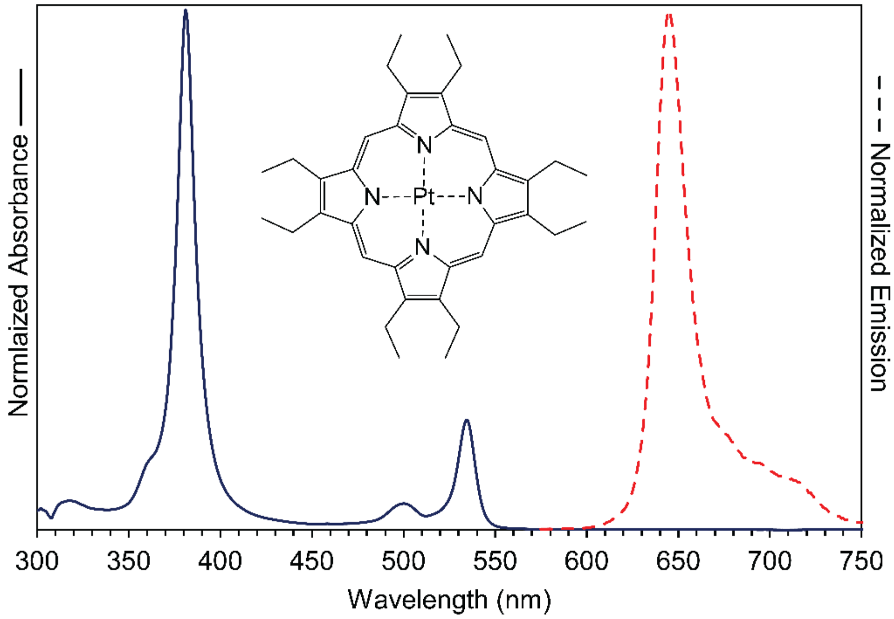

Figure S6. Absorption (solid blue) and emission (dashed red) spectra of PtOEP in 2-phenoxyethyl acrylate. The absorbance maxima are 381 and 535 nm and phosphorescence maximum is 645 nm (= 1.91 eV).

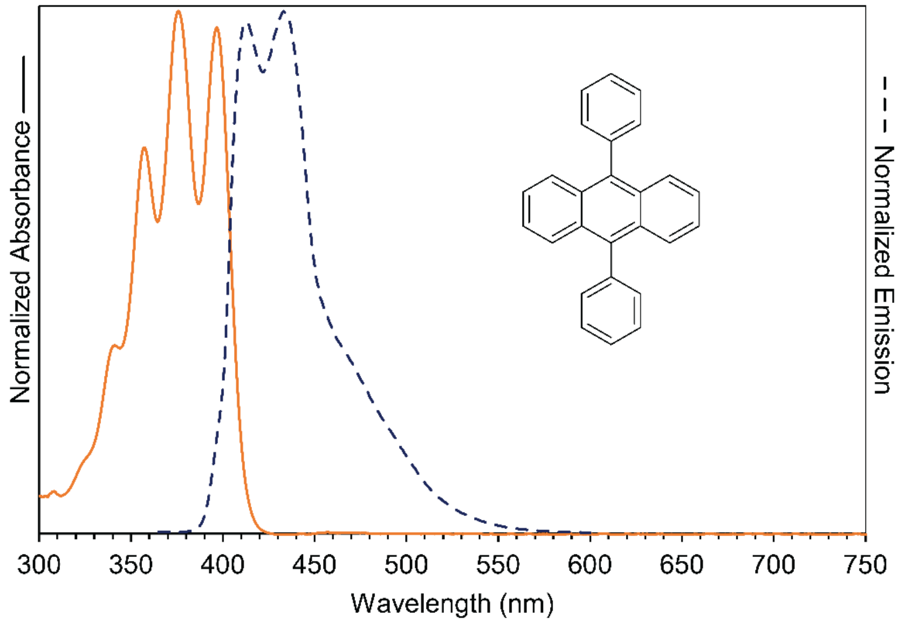

Figure S7. Absorption (solid yellow) and emission (dashed blue) spectra of DPA in 2-phenoxyethyl acrylate. The absorbance maximum occurs at 376 nm and phosphorescence maxima at 413 and 433 nm.

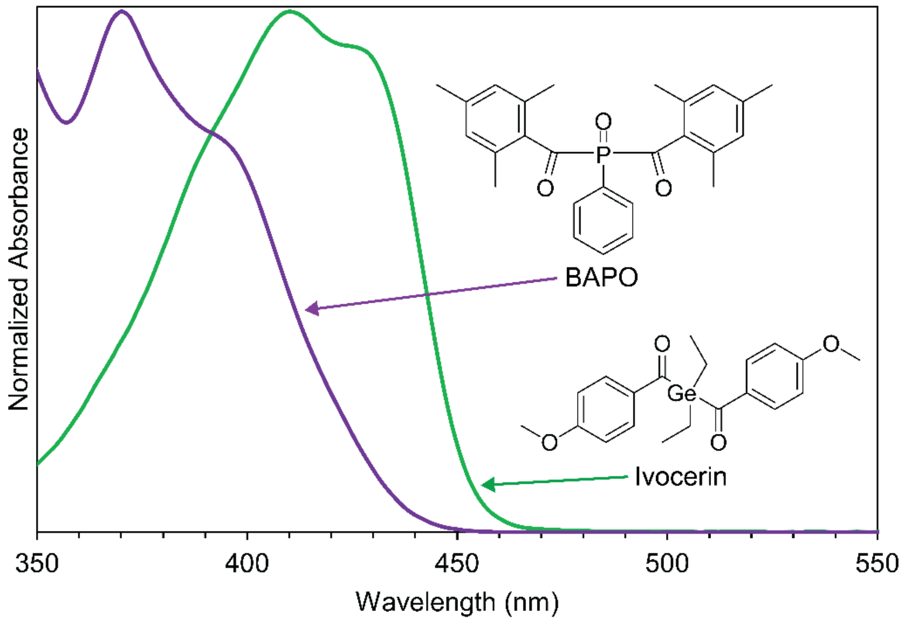

Figure S8. Absorbance spectra of BAPO and Ivocerin® in 2-phenoxyethyl acrylate.

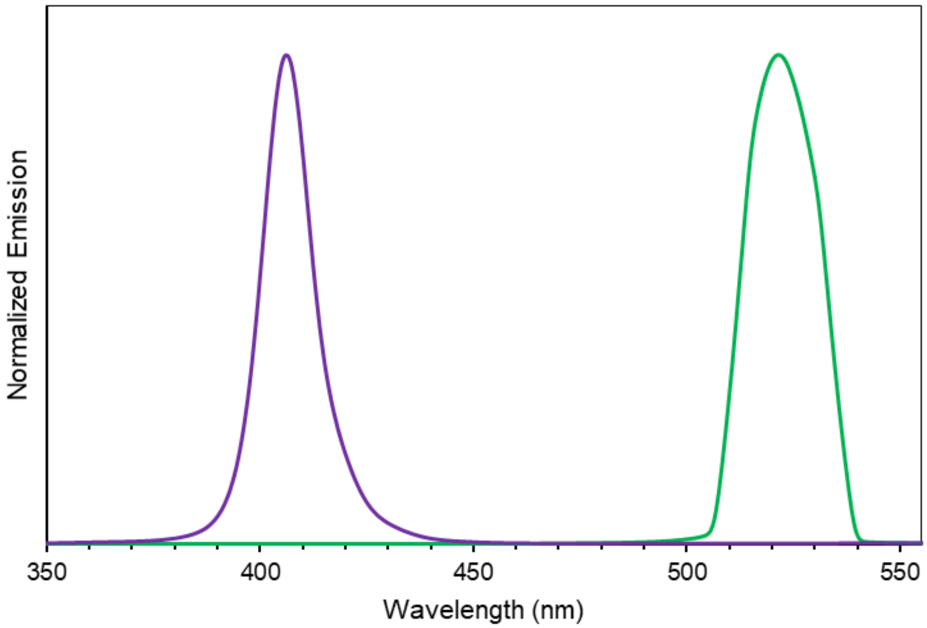

Figure S9. Normalized emission spectra of 405 nm LED (purple) and 525 nm LED equipped with a 525×25 nm bandpass filter (green).

RT-FTIR Monitoring of Photopolymerization Kinetics

Photopolymerization kinetics was monitored via the loss of the monomer C=C stretching band (802-818 cm^{-1}) for ATR and (6105-6214 cm^{-1}) for transmission mode FTIR. Maximum monomer conversion (ρ_{max}) is the total conversion reached after 110 seconds of irradiation found as an average of the final 2 seconds; rate of polymerization (r_p) is a linear fit of points between 4 seconds and the time required to reach a monomer conversion of $\frac{\rho_{max}}{5}$; inhibition time (t_{inh}) is the time required to pass 2% monomer conversion after the LED is turned on.

Monomer Selection. Different monomers were examined via ATR-FTIR irradiating with a 525 nm LED (10 mW/cm²) under inert conditions. The photosystem was comprised of PtOEP (0.005 mol%), DPA (0.01 mol%), and BAPO (0.50 mol%) (**Figure S10 and Table S1**).

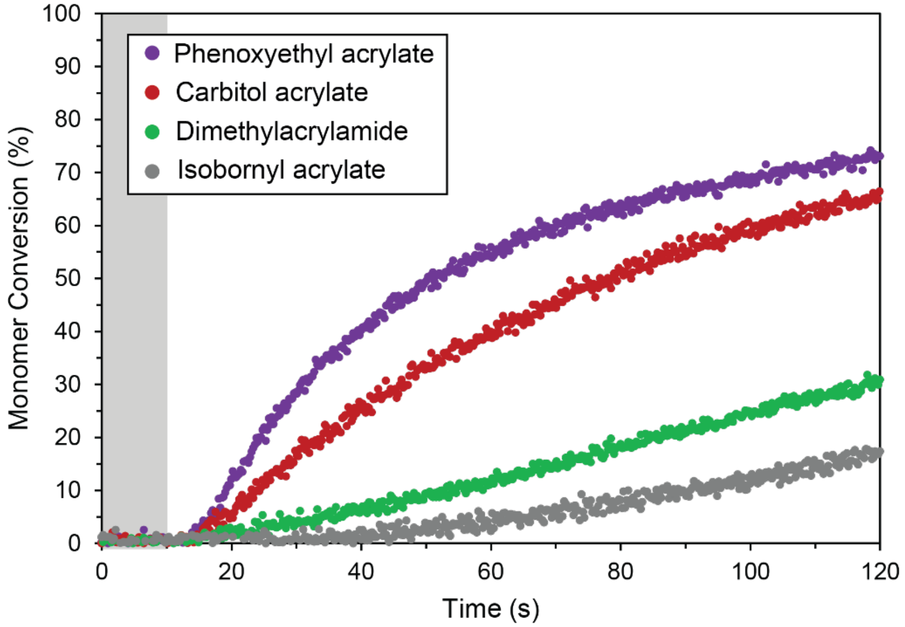

Figure S10. PtOEP (0.005 mol%), DPA (0.01 mol%), and BAPO (0.50 mol%) in different monomers irradiated with a 525 nm LED at 10 mW/cm². Measured via FTIR-ATR under argon atmosphere.

Table S1. Tabulated rates and conversions for different monomers. Values are averages from triplicate measurements (or more) with ±1 standard deviation from the mean.

Monomer	r_p (mM/s)	ρ_{max} (%)
2-phenoxyethyl acrylate	178.6 ± 8.1	81.2 ± 3.1
Carbitol acrylate	55.8 ± 7.1	64.7 ± 4.7
Dimethylacrylamide	25.8 ± 3.5	29.8 ± 1.7
Isobornyl acrylate	9.3 ± 0.9	17.7 ± 0.9

r_p = rate of polymerization post-induction period; ρ_{max} = maximum monomer conversion reached after 110 seconds of irradiation.

Upconversion Quantum Yield

Optical upconversion measurements (**Figure S13**) were performed by collecting the emission from the front face of the 1 mm path length cuvette at approximately a 45-degree angle due to the high optical densities of the resins. The upconversion samples were thoroughly purged of oxygen by bubbling with nitrogen for at least 30 minutes and adding 30 mM of oleic acid as an oxygen scavenger[S2] to improve the stability of the sample over the long measurement times. The upconversion quantum yield (Φ_{UC}) in 2-phenoxyethyl acetate was determined using Rhodamine 6G in ethanol (fluorescence quantum yield, Φ_{ref}, of 0.95)[S3] as a reference and using Equation S1:[S4]

$$\Phi_{\mathrm{UC}} = \Phi_{\mathrm{ref}} \left(\frac{1-10^{-A_{\mathrm{ref}}}}{1-10^{-A_{\mathrm{UC}}}}\right) \left(\frac{I_{\mathrm{UC}}}{I_{\mathrm{ref}}}\right) \left(\frac{\eta_{\mathrm{UC}}}{\eta_{\mathrm{ref}}}\right)^2 \quad \text{(S1)}$$

Here, A_{ref} and A_{UC} are the absorbances of the reference and upconversion sample at 532 nm, I_{ref} and I_{UC} are the integrated emission spectra of the reference (500 to 720 nm) and upconversion fluorescence (380 to 530 nm), and η_{ref} and η_{UC} are the refractive indices of the solvents (1.51 for 2-phenoxyethyl acetate and 1.36 for ethanol), respectively. The Rhodamine 6G sample was prepared in a 1 cm pathlength cuvette at a concentration that gave a maximum absorbance of 0.1 to minimize the inner filter effect.

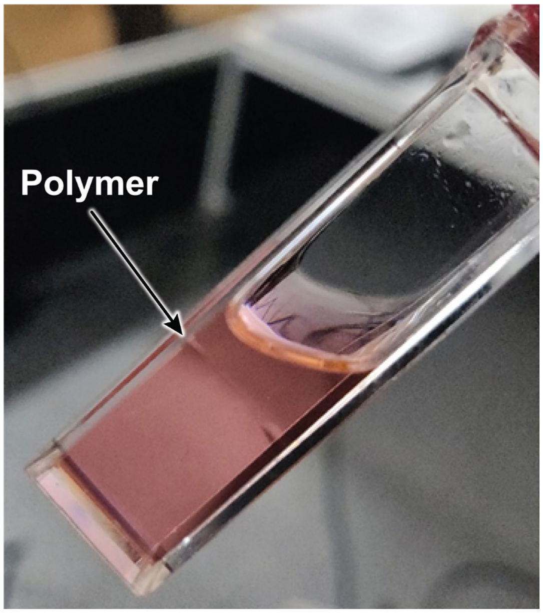

Figure S11. Image of the upconversion mixture without BAPO, showing polymer formation occurred after high-intensity (1000 mW/cm^2) 532 nm laser excitation over the course of 2 minutes.

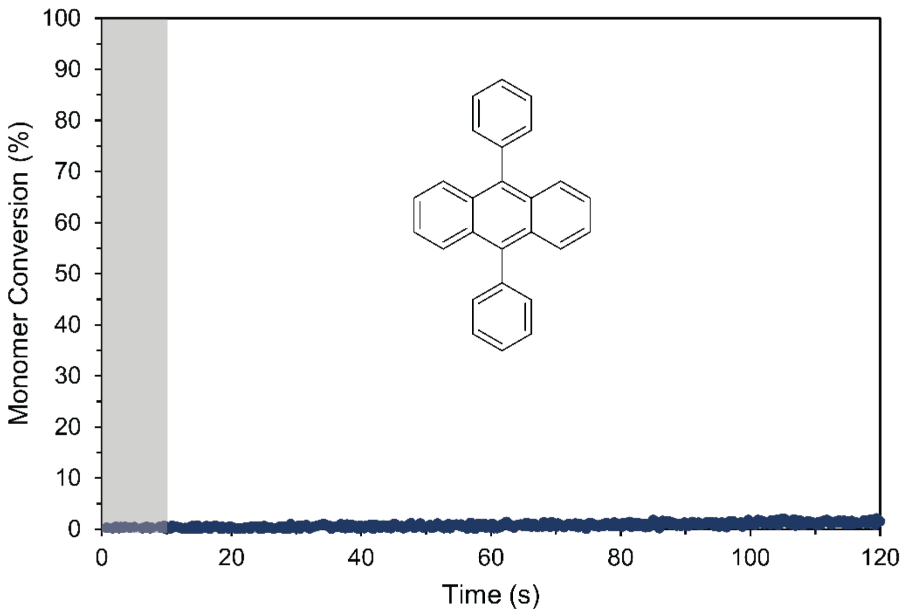

Figure S12. RT-FTIR-ATR measurement of resin containing only DPA (0.10 mol%) in 2-phenoxyethyl acrylate irradiated with a 405 nm LED (50 mW/cm²) under an argon atmosphere, showing no considerable polymerization in this timeframe.

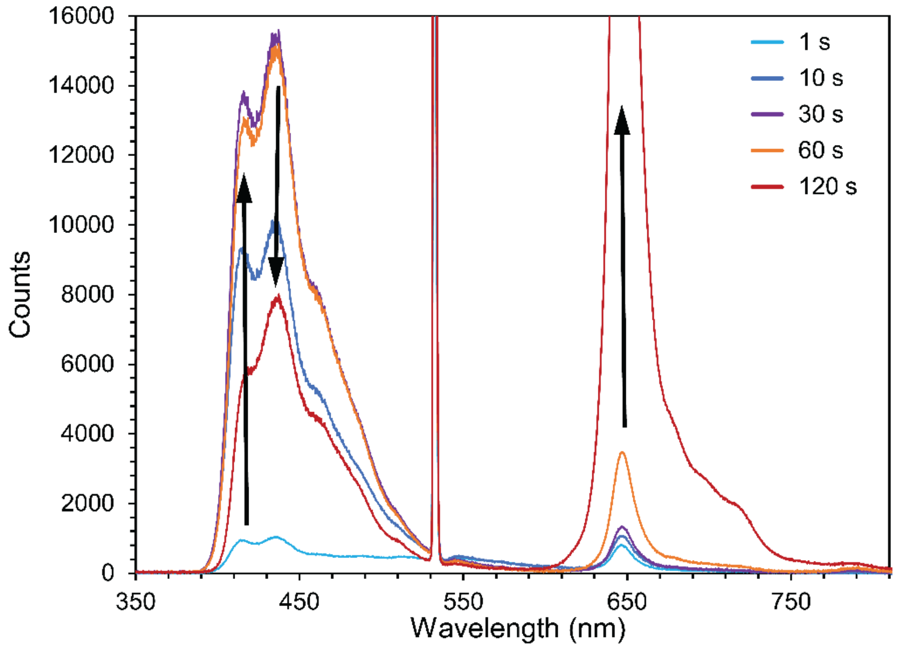

Figure S13. Emission spectra of the upconversion system without BAPO in the acrylate monomer at time points between 1 and 120 s with an excitation wavelength of 532 nm at an intensity of 1000 mW/cm^2.

Transient Absorption Spectroscopy

The triplet lifetimes of DPA and PtOEP were measured via transient absorption spectroscopy upon irradiation with a 532 nm pulsed laser. PtOEP phosphorescence decay was recorded at the peak wavelength of 645 nm. DPA triplet decay was recorded by monitoring the transient absorption of PtOEP sensitized DPA at 475 nm.[S5] The resulting transient photoluminescence decays were fitted with a single exponential function in Mathworks MATLAB R2022B software to obtain the triplet lifetimes of PtOEP.

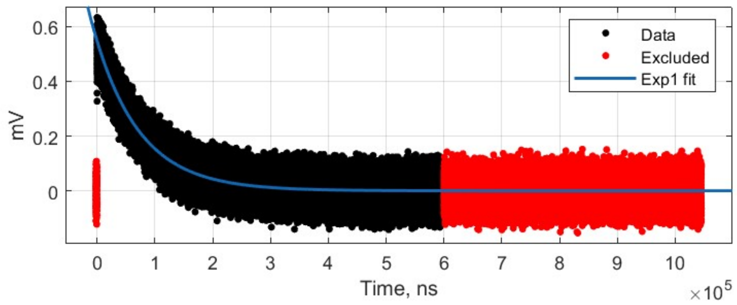

Figure S14. PtOEP phosphorescence decay in 2-phenoxyethyl acrylate without DPA monitored at 645 nm fitted with a single exponential function that yields a triplet lifetime of 78.7μs. Pulse fluence was 0.40 μJ/cm^2.

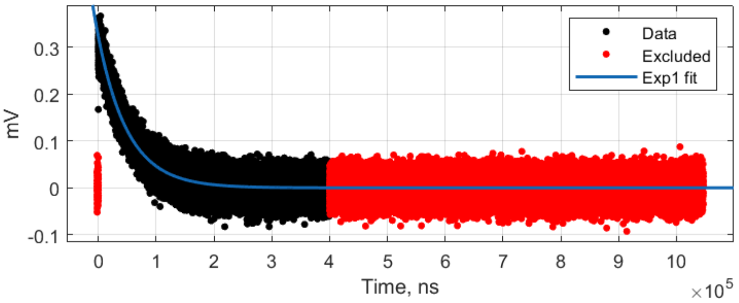

Figure S15. PtOEP phosphorescence decay in 2-phenoxyethyl acetate without DPA monitored at 645 nm fitted with a single exponential function that yields a triplet lifetime of 51.5 μs. Pulse fluence was 0.40 μJ/cm^2.

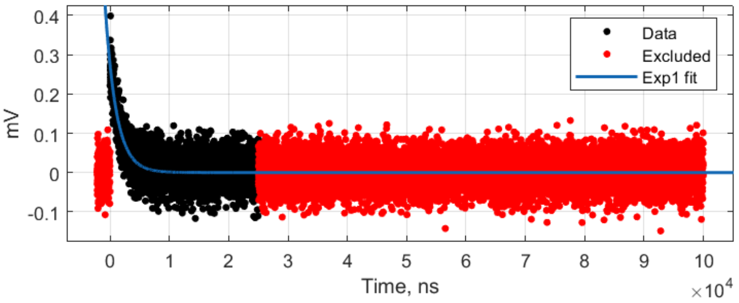

Figure S16. PtOEP phosphorescence decay in 2-phenoxyethyl acrylate with DPA monitored at 645 nm fitted with a single exponential function that yields a triplet lifetime of 1.88 μs. Pulse fluence was 0.40 μJ/cm^2.

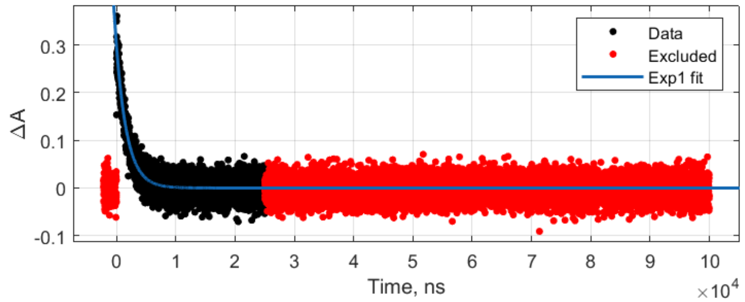

Figure S17. PtOEP phosphorescence decay in 2-phenoxyethyl acetate with DPA monitored at 645 nm fitted with a single exponential function that yields a triplet lifetime of 1.85 µs. Pulse fluence was 0.40 µJ/cm^2.

The triplet decay traces of DPA were measured under two low fluences of 0.23 µJ/cm^2 and 0.31 µJ/cm^2. The resulting decays were analyzed to yield both the spontaneous triplet decay rate ($k_{\mathrm{An}}^{\mathrm{T}}$) and the fraction ($\beta$) of triplets that undergo second-order decay, in this case via TTA. The analysis is based on solving the following differential rate equation that governs the triplet decay:[S6,S7]

$$\frac{d[An_T]}{dt} = -k_{\mathrm{An}}^{\mathrm{T}}[An_T] - k_{TTA}[An_T]^2 \tag{S2}$$

This equation has the following analytic solution:

$$\frac{[An_T]}{[An_T]_0} = \frac{1-\beta}{\exp(k_{\mathrm{An}}^{\mathrm{T}}t)-\beta}, \tag{S3}$$

where $\beta = \frac{k_{TTA}[An_T]_0}{k_{\mathrm{An}}^{\mathrm{T}}-k_{TTA}[An_T]_0}$ and equates to the fraction of annihilator triplets undergoing TTA at time zero. Thus, fitting equation S3 to the annihilator triplet decays yields both the spontaneous triplet decay rate and the fraction of annihilating triplets at time zero, which can be used to qualitatively compare the rate of TTA between the acetate and acrylate systems. The fitting was performed on OriginLab Origin 2024 by globally sharing $k_{\mathrm{An}}^{\mathrm{T}}$ between the decays measured in each solvent at different fluences while allowing β to float. These normalized decays and the respective fits are shown in **Figures S18** (acrylate) and **S19** (acetate). As expected, β increases in both solvents as fluence increases. More notably, β is significantly higher in acrylate, indicating that the rate of TTA is considerably faster.

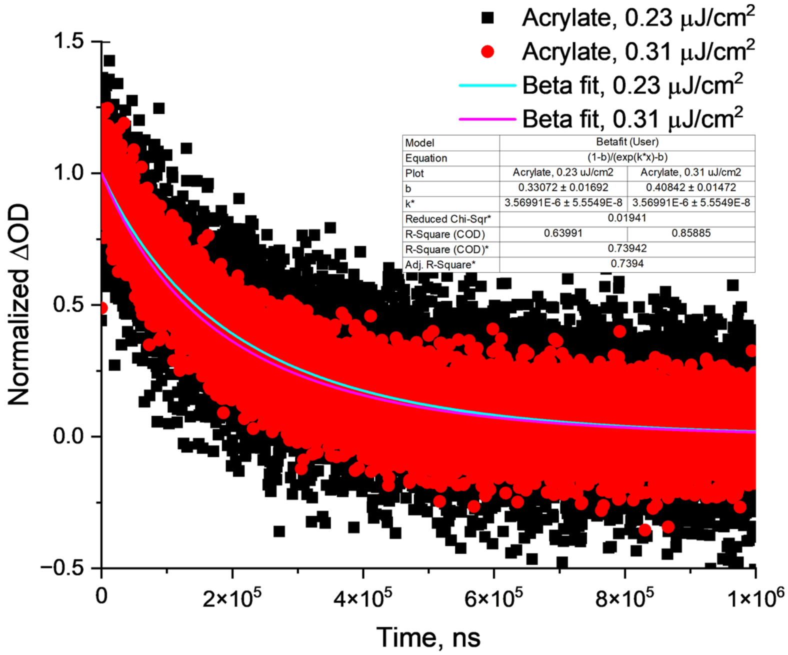

Figure S18. Normalized DPA triplet decay in 2-phenoxyethyl acrylate, monitored at 475 nm, fitted with equation S3, which yields $k_{\mathrm{An}}^{\mathrm{T}}$ = 3,570 s^{-1}. The value of β reaches 0.41 under the higher fluence.

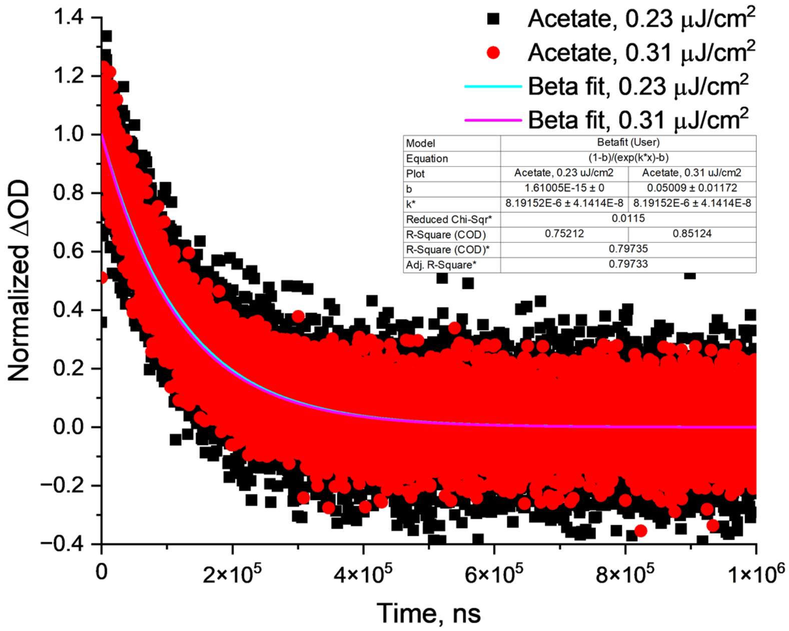

Figure S19. Normalized DPA triplet decay in 2-phenoxyethylacetate, monitored at 475 nm, fitted with equation S3, which yields $k_{\mathrm{An}}^{\mathrm{T}}$ = 8,190 s^{-1}. The value of β reaches 0.05 under the higher fluence.

Table S2. Time-resolved spectroscopic results of the TTA-UC system in acrylate and acetate.

	**Triplet Lifetime (µs)**	
	2-phenoxyethyl acrylate	2-phenoxyethyl acetate
**PtOEP Lifetime**	78.7	51.5
**PtOEP Lifetime in presence of DPA**	1.88	1.85
**DPA Lifetime**	280	122

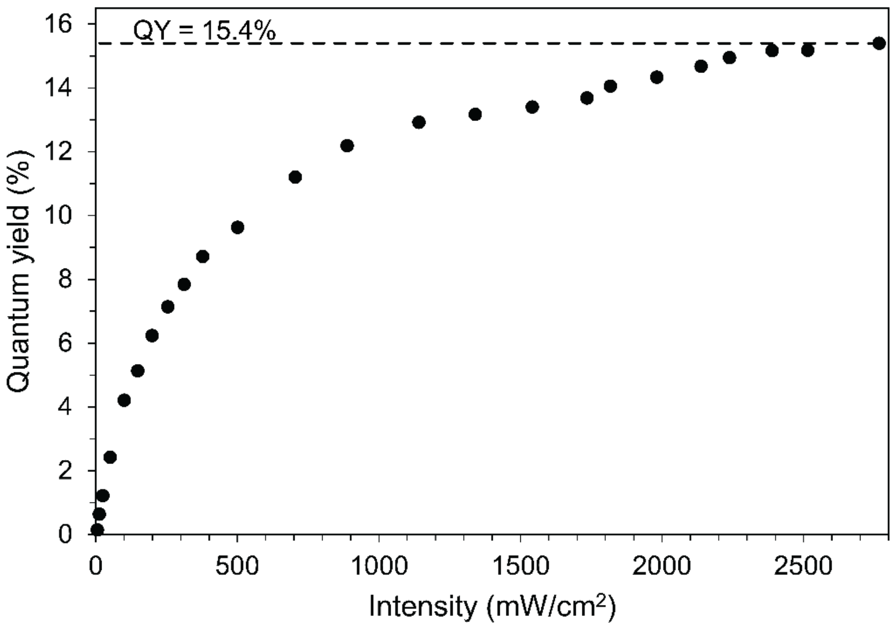

Figure S20. Quantum yield determination of the upconversion system in proxy solvent (2-phenoxyethyl acetate) using the upconverted emission measurement setup (**Figure S1**).

Fluorescence Lifetime
The fluorescence decays of DPA directly excited by a 375 nm laser were recorded using time-correlated single photon counting (**Figure S21**). The fluorescence decays were fitted with a single exponential function deconvoluted with the instrument response function in the Horiba DAS6 software and the fit residuals were inspected to evaluate the goodness of the fit. We find that the emission lifetime of DPA in 2-phenoxyethyl acrylate and 2-phenoxyethyl acetate is nearly identical (6.82 ns) indicating that DPA's S1 state is not quenched by 2-phenoxyethyl acrylate. For this reason, we hypothesize that charge transfer from DPA's triplet pair state prepared by TTA is responsible for photopolymerization in the absence of BAPO (**Figure S11**).

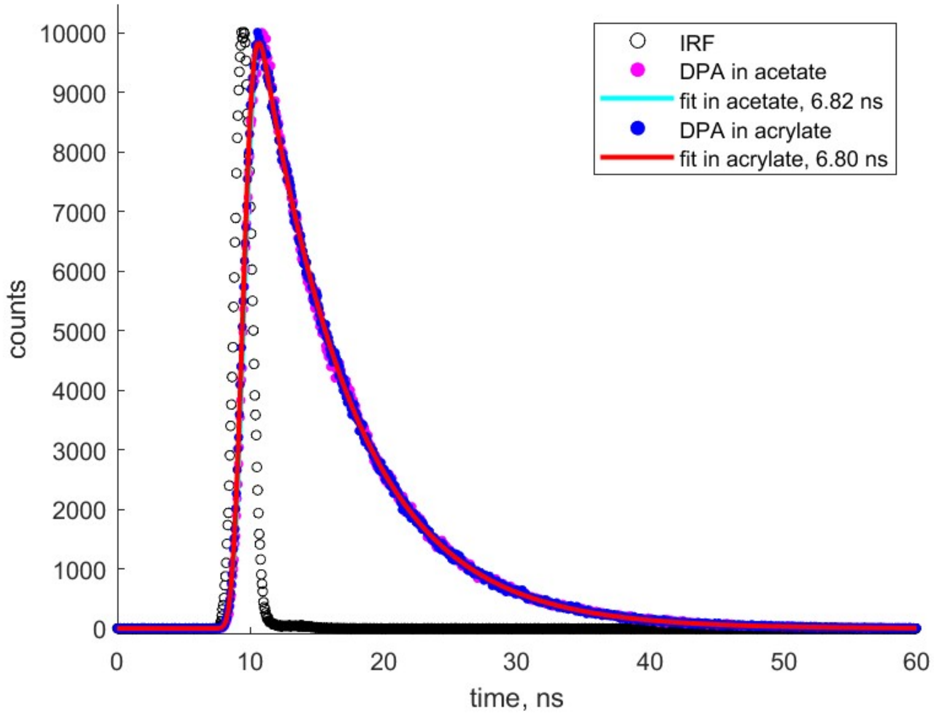

Figure S21. Fluorescence lifetime measurements of DPA in both acrylate and acetate after 375 nm excitation and corresponding fits to the data. IRF = instrument response function.

ATR-FTIR Characterization (Inert)

Photopolymerization optimization-1. The effect of photoinitiator (BAPO) concentration and light intensity on the polymerization kinetics were examined (**Figures S22-S23**).

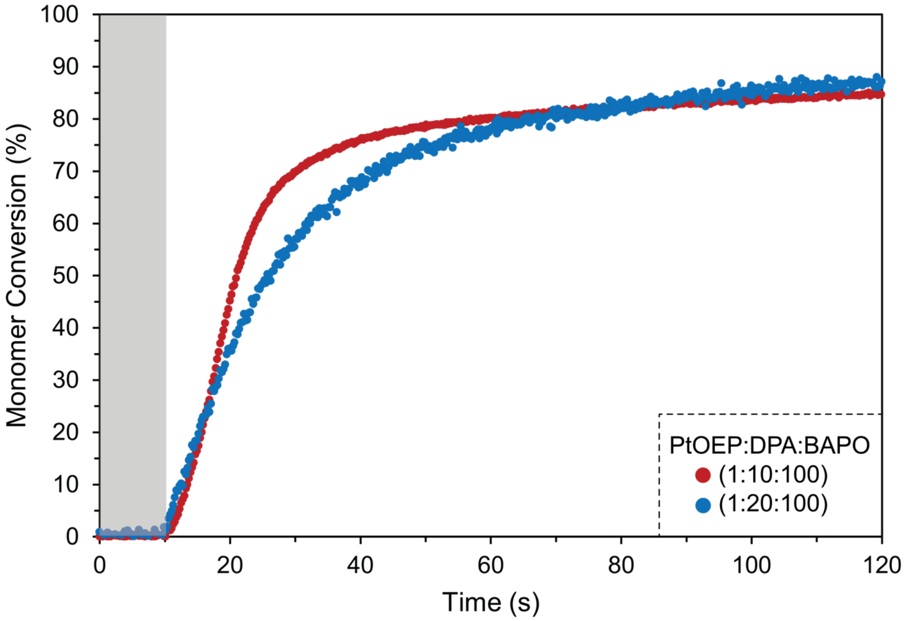

Figure S22. PtOEP:DPA:BAPO (1:20:100 ratio, 0.6, 12, 60 mM; blue) and PtOEP:DPA:BAPO (1:10:50 ratio, 0.6, 6, 30 mM, red) in 2-phenoxyethyl acrylate irradiated with a 525 nm LED at 10 mW/cm². Measured on FTIR-ATR under an argon atmosphere. Blue trace: r_p = 201 mM/s, ρ_{max} = 86.9 % after 110 seconds of irradiation. Red trace: r_p = 240 mM/s, ρ_{max} = 84.7 % after 110 seconds of irradiation.

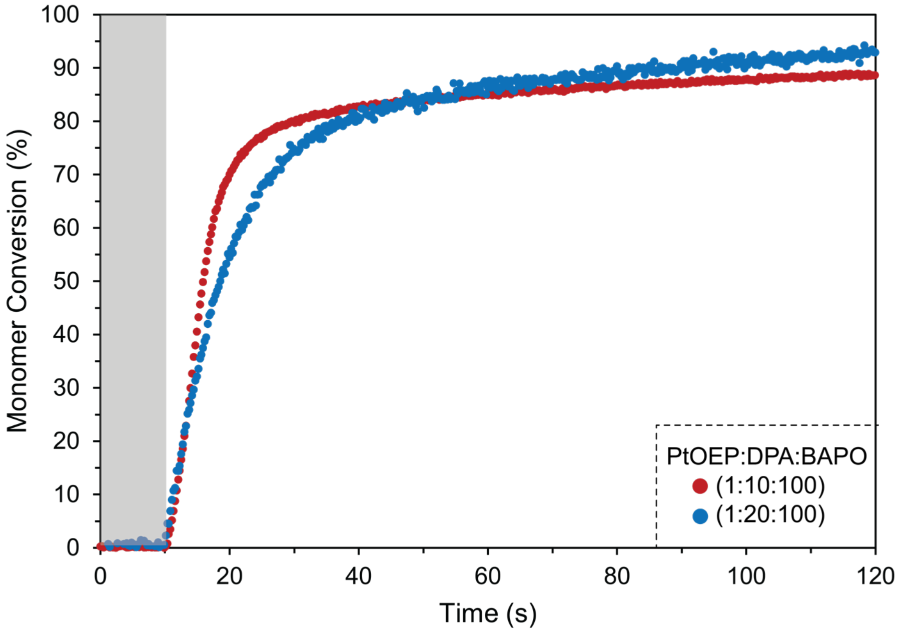

Figure S23. PtOEP:DPA:BAPO (1:20:100 ratio, 0.6, 12, 60 mM; blue) and PtOEP:DPA:BAPO (1:10:50 ratio, 0.6, 6, 30 mM, red) in 2-phenoxyethyl acrylate irradiated with a 525 nm LED at 20 mW/cm². Measured on FTIR-ATR under an argon atmosphere. Blue trace: r_p = 362 mM/s, ρ_{max} = 92.8 % after 110 seconds of irradiation. Red trace: r_p = 452 mM/s, ρ_{max} = 88.7 % after 110 seconds of irradiation.

Table S3. Summary of photopolymerization kinetics of optimized resin as measured using RT-FTIR and provided in Fig. 4B. Resin composition: [PtOEP] = 0.6 mM; [DPA] = 6 mM; [BAPO] = 30 mM. Values are averages from triplicate measurements (or more) with ±1 standard deviation from the mean.

Conditions	[PtOEP] (μM)	PtOEP:DPA:BAPO ratio	I_{ex} (mW/cm^2)	r_p (mM/s)	ρ_{max} (%)	t_{inh} (s)
Inert	600	1:10:50	50	901 ± 41	93	<0.1
Inert	600	1:10:50	5	109 ± 6.4	81	<0.1
Ambient	600	1:10:50	50	834 ± 34	92	0.9 ± 0.3
Ambient	600	1:10:50	5	85 ± 7	75	7.1 ± 0.5
Ambient	300	1:10:50	50	504 ± 25	87	2.0 ± 0.2
Ambient	300	1:10:50	5	56 ± 3	59	21 ± 1.0

I_{ex} = excitation intensity; r_p = rate of polymerization post-induction period; ρ_{max} = maximum monomer conversion reached after 110 seconds of irradiation; t_{inh} = inhibition time after turning the LED 'on' during which no monomer conversion occurs.

Photopolymerization controls. The effect of removing one or more of the photosystem components on the polymerization kinetics was examined (**Figures S24-S25**).

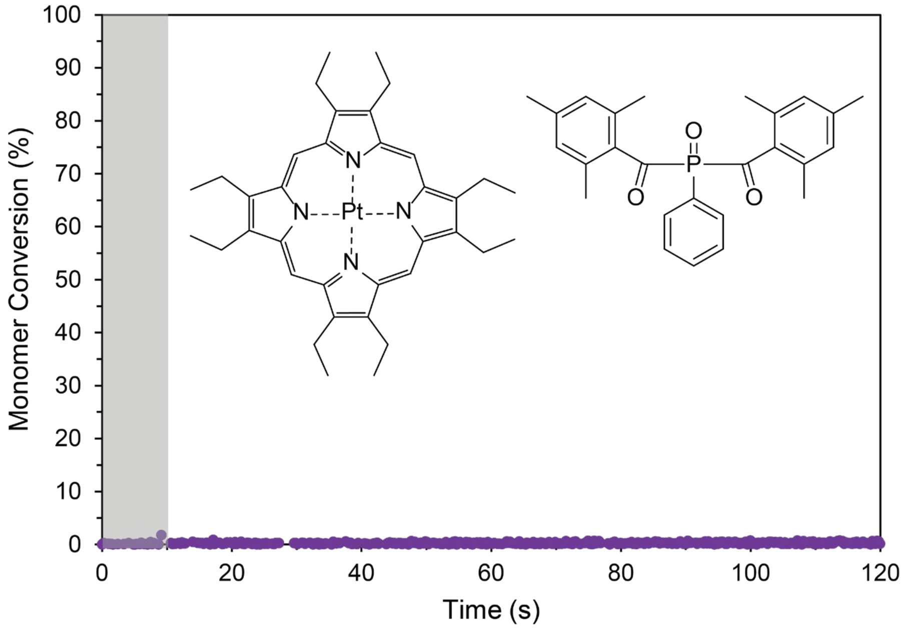

Figure S24. PtOEP (0.01 mol%) and BAPO (0.50 mol%) in 2-phenoxyethyl acrylate irradiated with a 525 nm LED at 50 mW/cm². Measured via FTIR-ATR under an argon atmosphere.

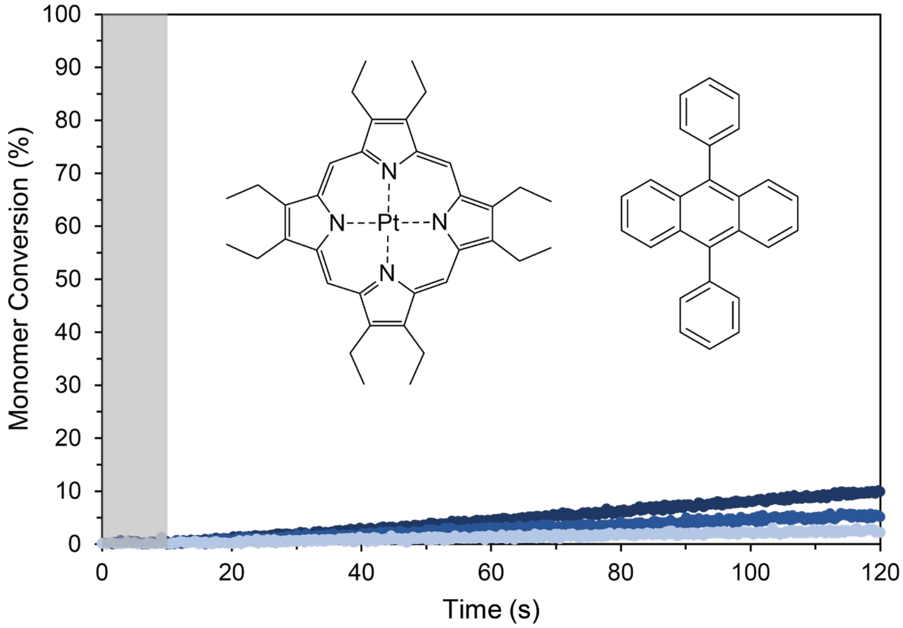

Figure S25. PtOEP (0.01 mol%) and DPA (0.10 mol%) in 2-phenoxyethyl acrylate irradiated with a 525 nm LED (10, 20 and 50 mW/cm²). Measured via FTIR-ATR under an argon atmosphere. At 50 mW/cm², $r_p = 5.3 \pm 0.5$ mM/s, $\rho_{max} = 9.7 \pm 0.2\%$ after 110 seconds of irradiation.

Alternate photoinitiator. The impact on polymerization kinetics due to replacing BAPO with Ivocerin® as an alternate photoinitiator was examined (**Figure S26**). See **Figure S8** for the UV-vis absorption spectrum of Ivocerin®.

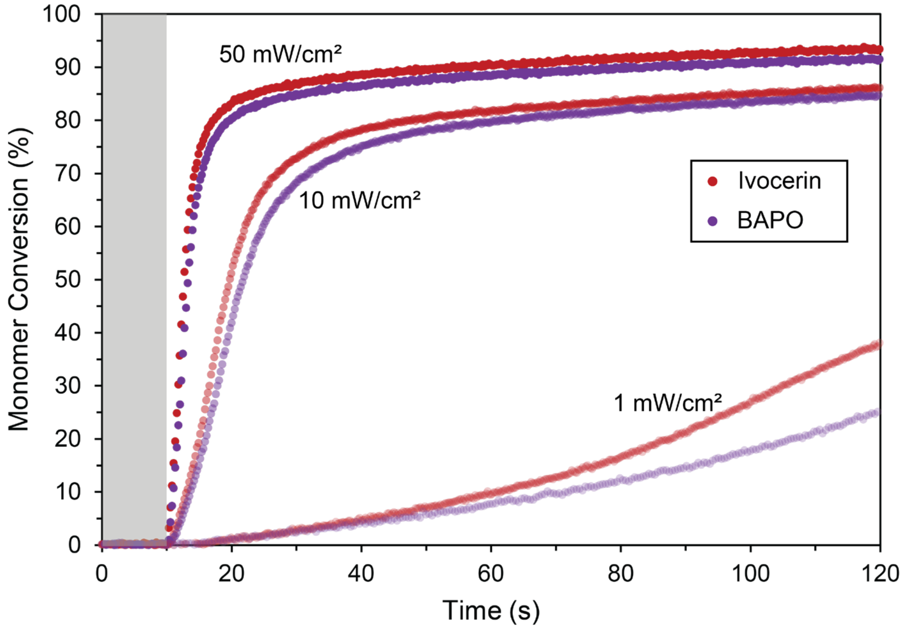

Figure S26. PtOEP (0.01 mol%) and DPA (0.10 mol%) in 2-phenoxyethyl acrylate with either Ivocerin® or BAPO (0.50 mol%, ~1 wt%) irradiated with a 525 nm LED (10, 20 and 50 mW/cm²). Measured via FTIR-ATR under an argon atmosphere.

Table S4. Summary of photopolymerization kinetics comparing Ivocerin® as an alternate photoinitiator to BAPO measured using RT-FTIR and provided in Fig. S26. Resin composition: [PtOEP] = 0.6 mM; [DPA] = 6 mM; [BAPO] or [Ivocerin®]= 30 mM.

PS	I_{ex} (mW/cm^2)	r_p (mM/s)	ρ_{max} (%)	t_{inh} (s)
BAPO	1	8.9	21.7	20.5
Ivocerin®	1	10.5	37.4	15.2
BAPO	10	227	84.6	1.4
Ivocerin®	10	261	86.0	1.2
BAPO	50	828	91.5	0.5
Ivocerin®	50	979	93.3	0.3

I_{ex} = excitation intensity; r_p = rate of polymerization post-induction period; ρ_{max} = maximum monomer conversion reached after 110 seconds of irradiation; t_{inh} = inhibition time after turning the LED 'on' during which no monomer conversion occurs.

Photopolymerization optimization-2. The influence of overall photosystem concentration and light intensity on polymerization kinetics was examined (**Figures S27-S30**).

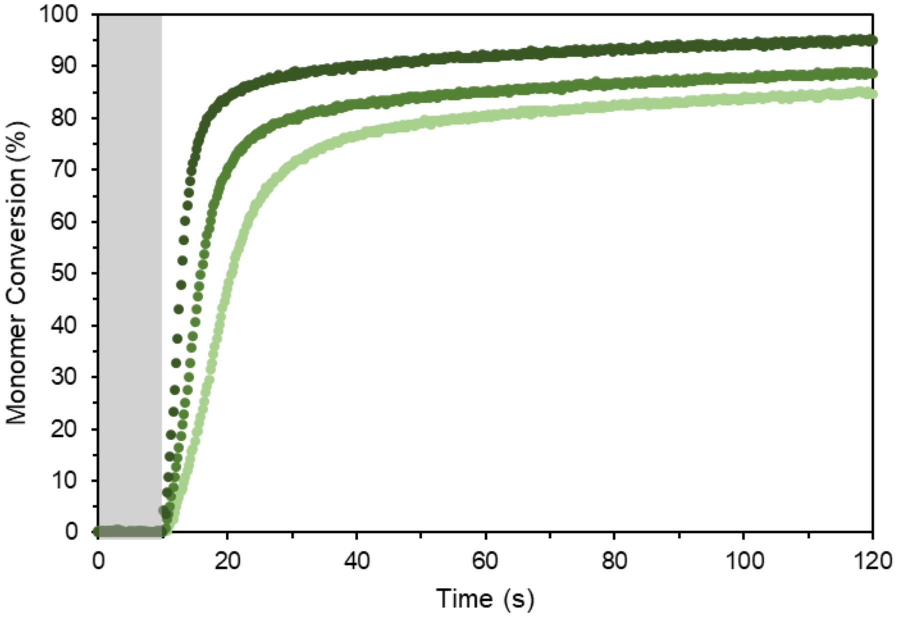

Figure S27. Highest concentration resin: ([PS] = 0.01 mol%, 0.6 mM) in a ratio of PtOEP:DPA:BAPO (1:10:50) in 2-phenoxyethyl acrylate irradiated with a 525 nm LED at 10, 20 and 50 mW/cm². Measured via FTIR-ATR under argon atmosphere.

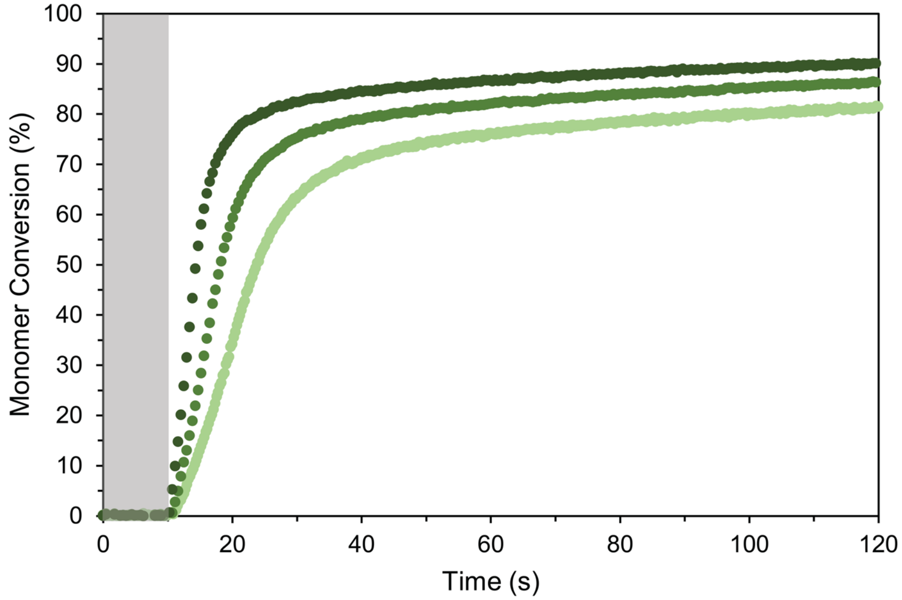

Figure S28. Resin diluted by 1/2: ([PS] = 0.005 mol%, 0.3 mM) in a ratio of PtOEP:DPA:BAPO (1:10:50) in 2-phenoxyethyl acrylate irradiated with a 525 nm LED at 10, 20 and 50 mW/cm². Measured via FTIR-ATR under argon atmosphere.

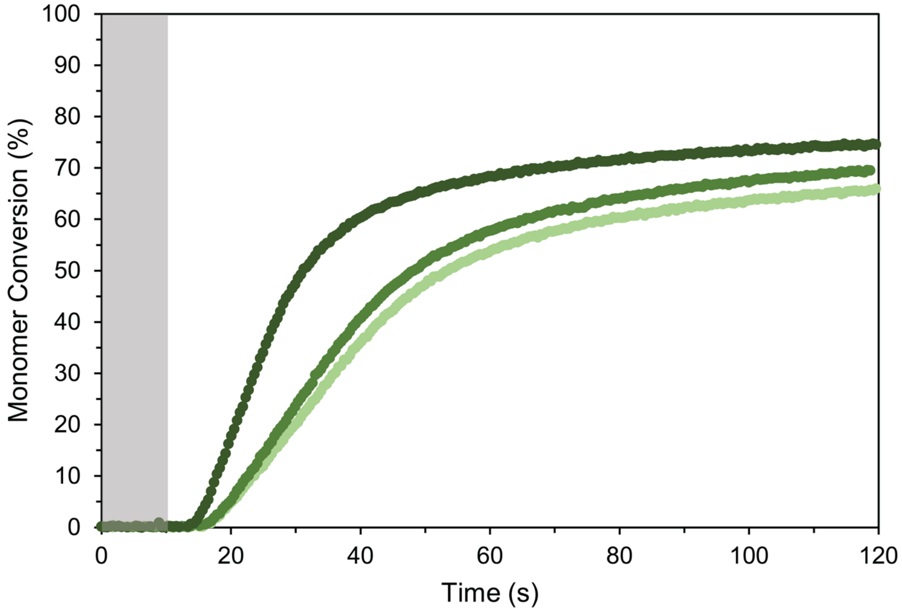

Figure S29. Resin diluted by 1/10: ([PS] = 0.001 mol%, 0.06 mM) in a ratio of PtOEP:DPA:BAPO (1:10:50) in 2-phenoxyethyl acrylate irradiated with a 525 nm LED at 10, 20 and 50 mW/cm². Measured via FTIR-ATR under an argon atmosphere.

Table S5. Summary of photopolymerization optimization-2 kinetics measured using RT-FTIR and provided in Fig. S26-S29. The PtOEP:DPA:BAPO ratio remained constant at 1:10:50.

[PtOEP] (µM)	I_{ex} (mW/cm^2)	r_p (mM/s)	ρ_{max} (%)	t_{inh} (s)
600	10	248	84.9	1.2
600	20	448	88.7	0.5
600	50	902	95.0	< 0.2
300	10	202	81.3	1.4
300	20	348	86.3	0.9
300	50	637	90.0	0.4
60	10	85.1	65.9	8.0
60	20	99.7	69.4	7.6
60	50	191	74.7	4.9

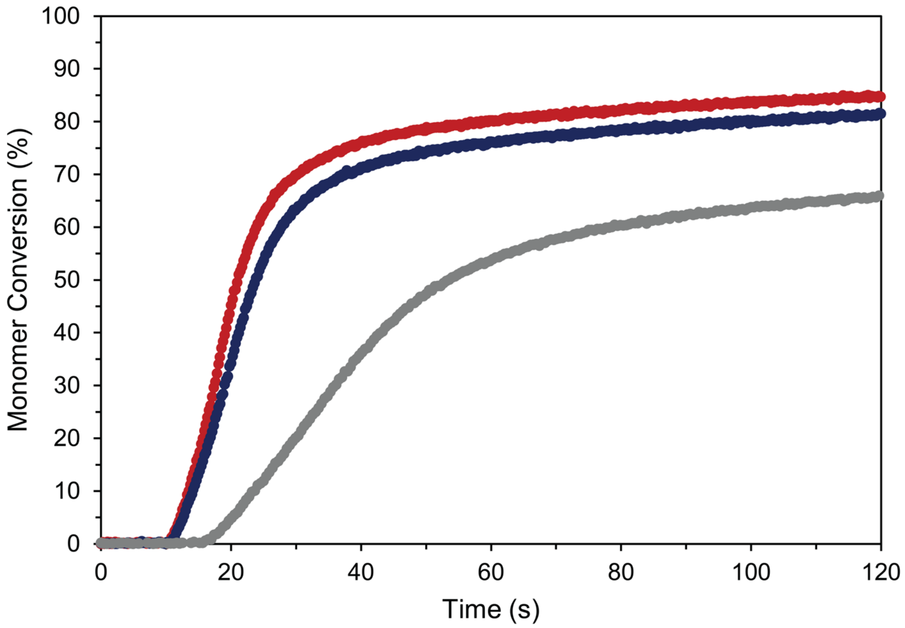

Figure S30. Comparison figure overlaying the three different photosystem ratios irradiated with a 525 nm LED at 10 mW/cm². A [PtOEP] = 0.01, 0.005, and 0.001 mol% (red, blue, and grey, respectively) were used with a ratio of PtOEP:DPA:BAPO (1:10:50) in 2-phenoxyethyl acrylate. Samples were measured via FTIR-ATR under an argon atmosphere.

Relationship between r_p and I_{ex} for TTA-UC to Type I. Using the optimized resin, photopolymerizations were conducted at several different intensities between 0.1 mW/cm^2 and 200 mW/cm^2. The complete data is provided in **Figures 5A** and **5B** in the main manuscript, with each different region individually plotted with fits below in **Figures S31-S33**.

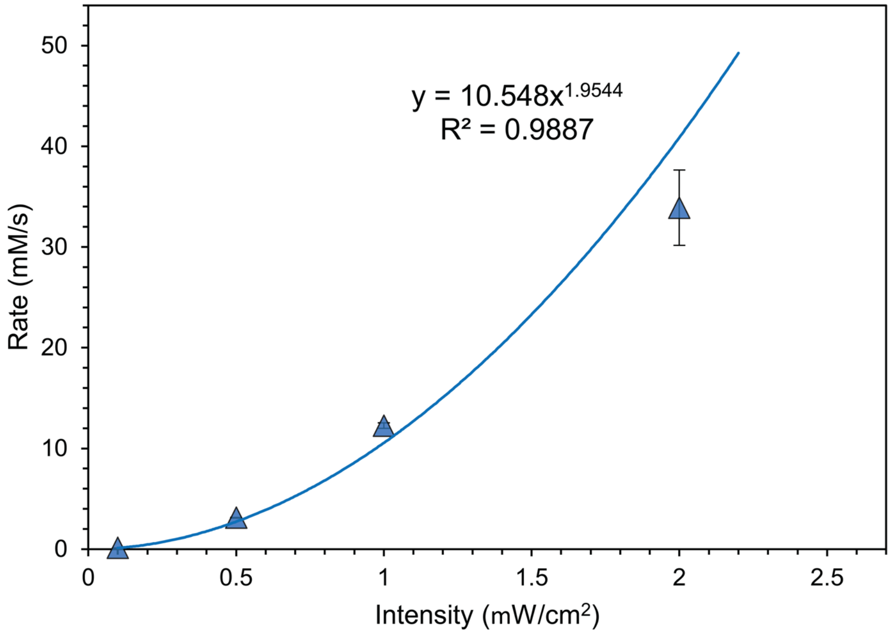

Figure S31. Upconversion resin comprised of PtOEP (0.01 mol%), DPA (0.10 mol%), and BAPO (0.50 mol%) in 2-phenoxyethyl acrylate. Sample irradiated with a 525 nm LED and monitored via FTIR-ATR under an argon atmosphere. The initial rate of polymerization was plotted against excitation intensity within the superlinear regime between 0.1 and 2 mW/cm^2.

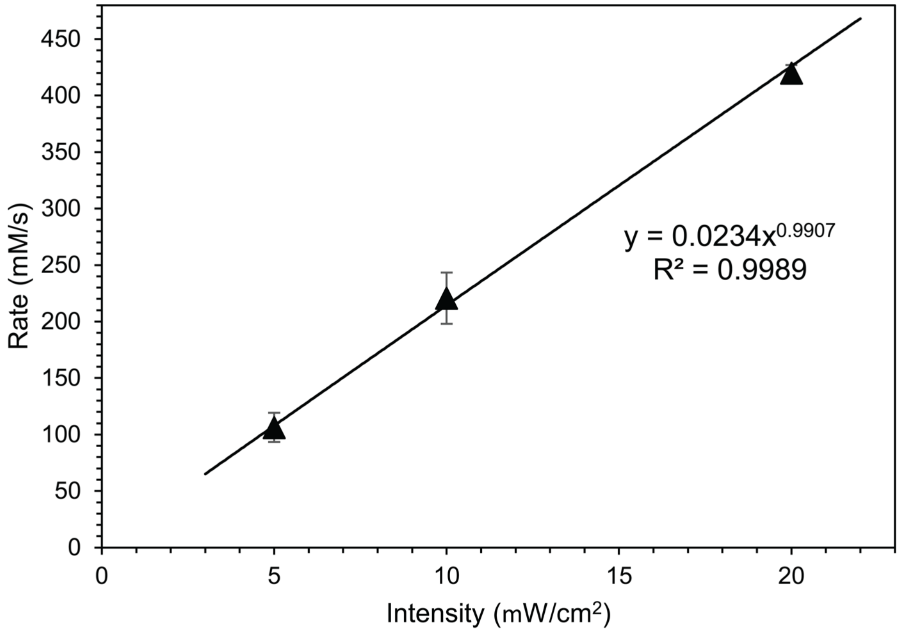

Figure S32. Upconversion resin comprised of PtOEP (0.01 mol%), DPA (0.10 mol%), and BAPO (0.50 mol%) in 2-phenoxyethyl acrylate. Sample irradiated with a 525 nm LED and monitored via FTIR-ATR under an argon atmosphere. The initial rate of polymerization was plotted against intensity within the linear regime between 5 and 20 mW/cm^2.

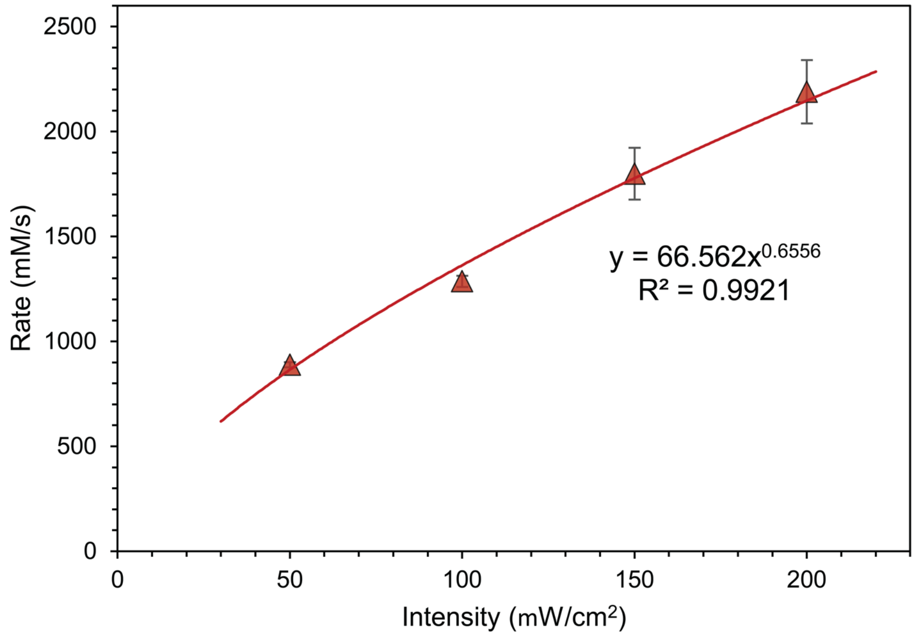

Figure S33. Upconversion resin comprised of PtOEP (0.01 mol%), DPA (0.10 mol%), and BAPO (0.50 mol%) in 2-phenoxyethyl acrylate. Sample irradiated with a 525 nm LED and monitored via FTIR-ATR under an argon atmosphere. The initial rate of polymerization was plotted against intensity within the sublinear regime between 50 and 200 mW/cm^2.

Table S6. Summary of TTA-UC to Type I photopolymerization kinetics as a function of light intensity measured using RT-FTIR and provided in Fig. 5A and 5B and Fig. S31-S33. The [PtOEP] was 600 µM and the ratio of PtOEP:DPA:BAPO was 1:10:50. Values are averages from triplicate measurements (or more) with ±1 standard deviation from the mean.

I_{ex} (mW/cm^2)	r_p (mM/s)	ρ_{max} (%)
0.1	0.106 ± 0.004	0.2
0.5	3.11 ± 0.05	5.0
1	12.3 ± 0.3	23.9
2	33.9 ± 3.7	67.2
5	106 ± 13	83.1
10	221 ± 23	86.1
20	420 ± 10	89.1
50	888 ± 12	92.8
100	1290 ± 27	94.2
150	1800 ± 120	96.8
200	2190 ± 150	98.0

Relationship between r_p and I_{ex} for Type I and Type II controls. Using the Type I and Type II control resins, photopolymerizations were conducted at several different intensities between 0.001 mW/cm^2 and 2 mW/cm^2. The complete data is provided in **Figure 5B** in the main manuscript and below in **Figures S34-S39**.

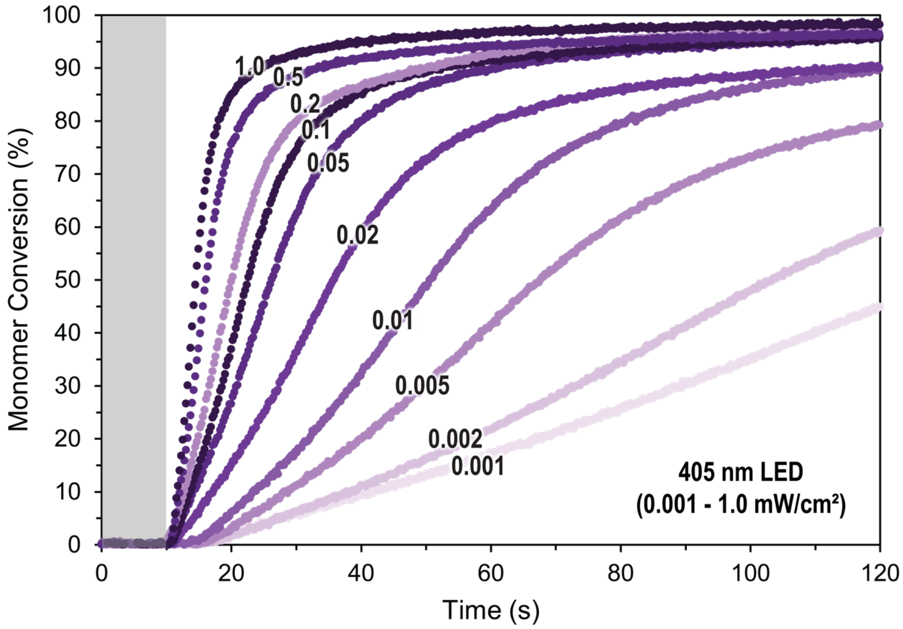

Figure S34. Representative traces of Type I resin comprising BAPO (0.50 mol%) in 2-phenoxyethyl acrylate and irradiated with a 525 nm LED between 0.001 and 1 mW/cm² while monitoring via FTIR-ATR under an argon atmosphere.

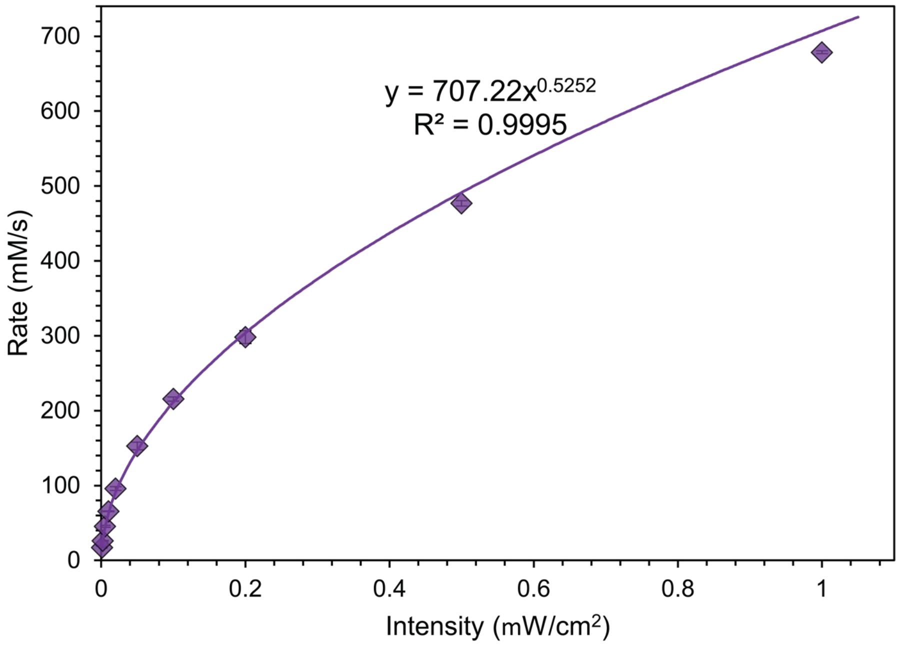

Figure S35. Plot of initial rate vs. excitation intensity for the Type I resin from 0.001-1 mW/cm². The trendline represents a power law fit to the data and shows a sublinear relationship between r_p and I_{ex}.

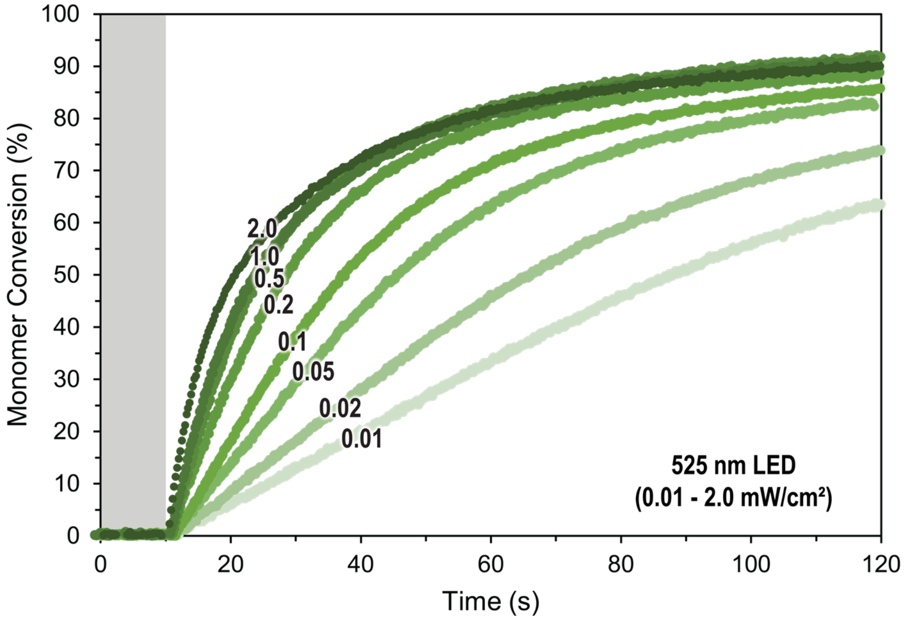

Figure S36. Representative traces of Type II resin comprising PtOEP (0.01 mol%), Borate V (0.05 mol%), and HNu-254 (0.50 mol%) in 2-phenoxyethyl acrylate. Samples were irradiated with a 525 nm LED between 0.01-2 mW/cm² while monitoring via FTIR-ATR under an argon atmosphere.

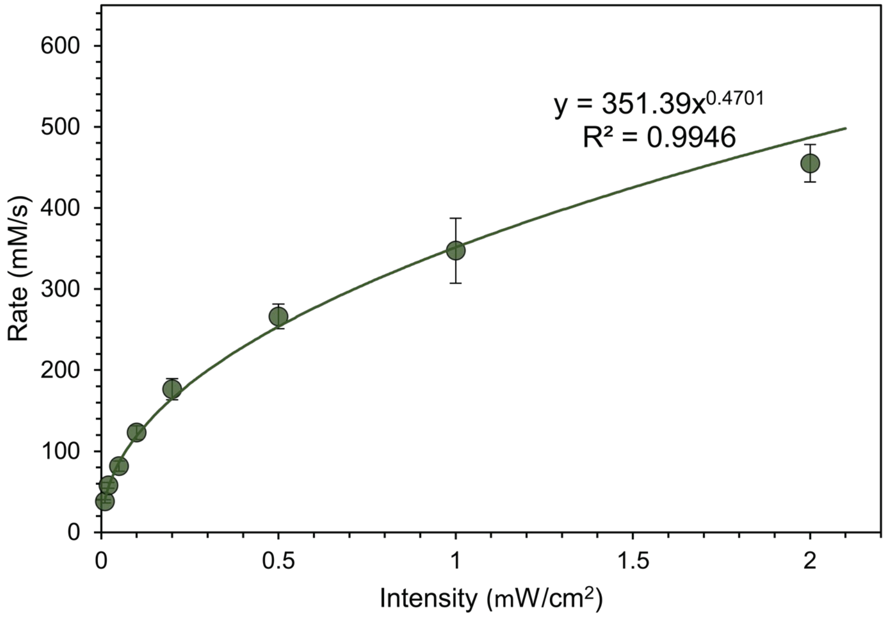

Figure S37. Plot of initial rate vs. excitation intensity for the Type I resin from 0.01-2 mW/cm². The trendline represents a power law fit to the data and shows a sublinear relationship between r_p and I_{ex}.

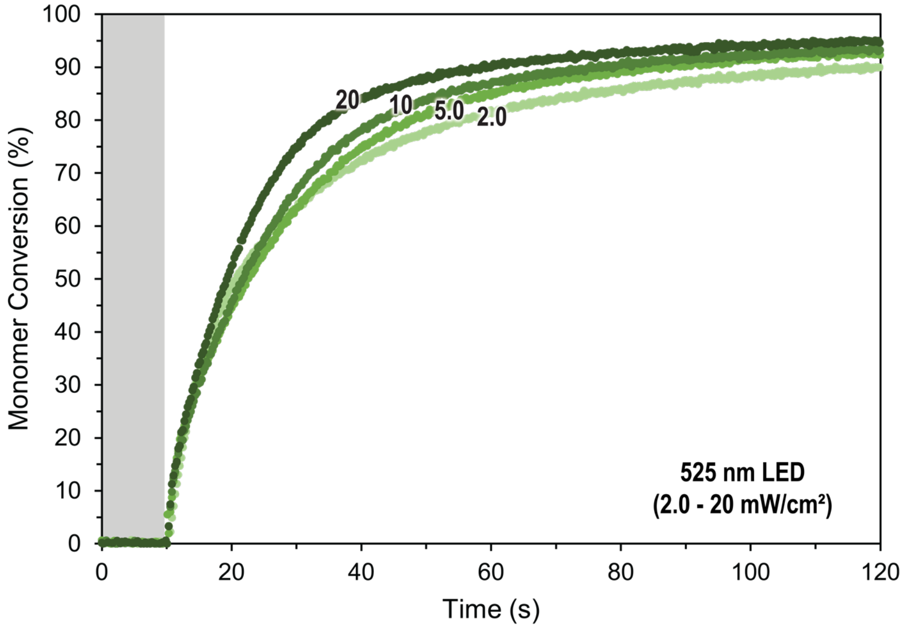

Figure S38. Representative traces of type II resins comprising PtOEP (0.01 mol%), Borate V (0.05 mol%), and HNu-254 (0.50 mol%) in 2-phenoxyethyl acrylate. Samples were irradiated with a 525 nm LED between 2 and 20 mW/cm² and monitored via FTIR-ATR under an argon atmosphere.

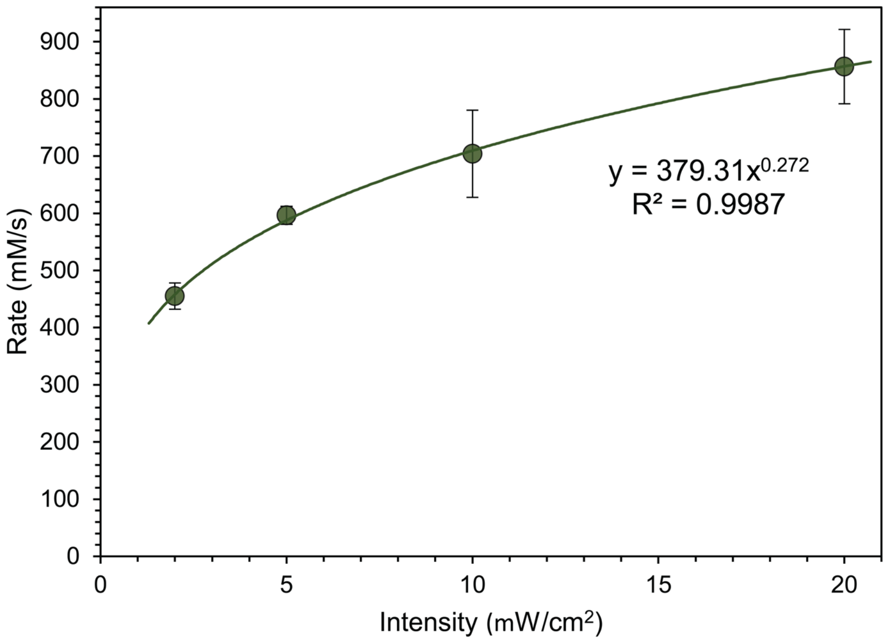

Figure S39. Plot of initial rate vs. excitation intensity for the Type II resin from 2-20 mW/cm². The trendline represents a power law fit to the data and shows a strong sublinear relationship between r_p and I_{ex} when operating at high light intensities. This is hypothesized to arise from competitive degradation that occurs at high I_{ex} values.

Table S7. Summary of Type I and Type II photopolymerization kinetics as a function of light intensity measured using RT-FTIR and provided in Fig. 5B and Fig. S34-S39. Values are averages from triplicate measurements (or more) with ±1 standard deviation from the mean.

Mechanism	I_{ex} (mW/cm^2)	r_p (mM/s)	ρ_{max} (%)
Type I	0.001	17.3 ± 1.1	41.3
	0.002	26.3 ± 0.6	56.0
	0.005	45.5 ± 1.2	79.9
	0.01	65.4 ± 0.4	87.4
	0.02	96.0 ± 2.6	89.4
	0.05	153 ± 5.1	94.1
	0.1	216 ± 2.8	94.5
	0.2	298 ± 8.5	96.0
	0.5	477 ± 3.5	96.1
	1	679 ± 2.0	97.4
Type II	0.01	38.2 ± 2.0	63.3
	0.02	58.0 ± 3.4	73.8
	0.05	81.6 ± 6.5	82.7
	0.1	123 ± 8.5	85.5
	0.2	177 ± 13	88.7
	0.5	266 ± 15	89.1
	1	347 ± 40	91.0
	2	455 ± 23	89.9
	5	596 ± 15	92.7
	10	704 ± 76	93.6
	20	856 ± 65	94.8

Shelf stability. The stability of the TTA-UC to Type I resin (**Figure S40**) and Type II resin (**Figure 41**) were assessed by monitoring photopolymerization kinetics using RT-FTIR-ATR under an argon atmosphere over the course of 45 days. Periodic aliquots were removed from samples stored at room temperature in the absence of light. Data in this section was used to create **Figure 5C** in the main text.

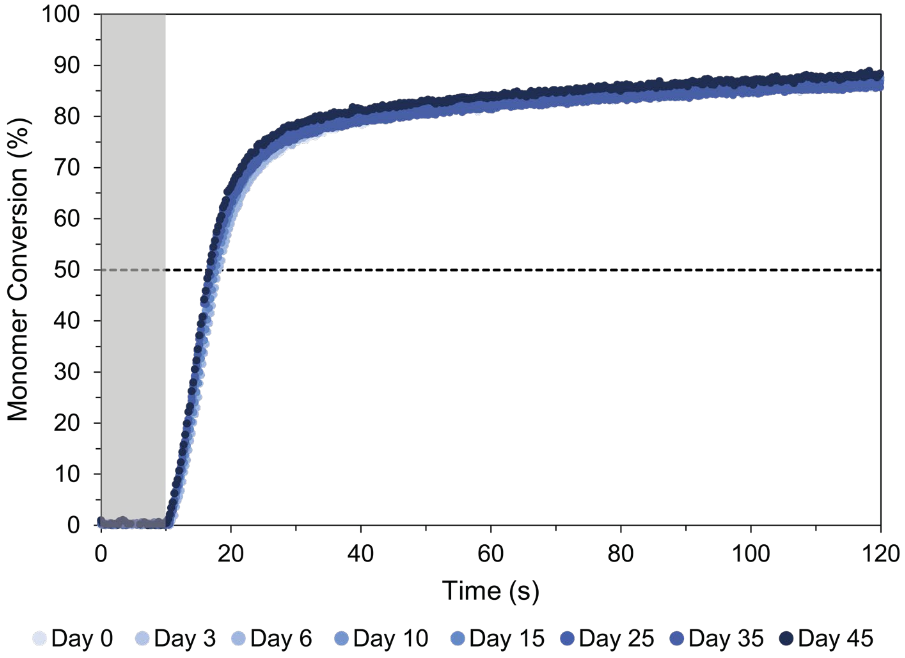

Figure S40. Plot of monomer conversion vs. time for the TTA-UC resin. The samples were degassed before each trial and then irradiated with a 525 nm LED at 20 mW/cm².

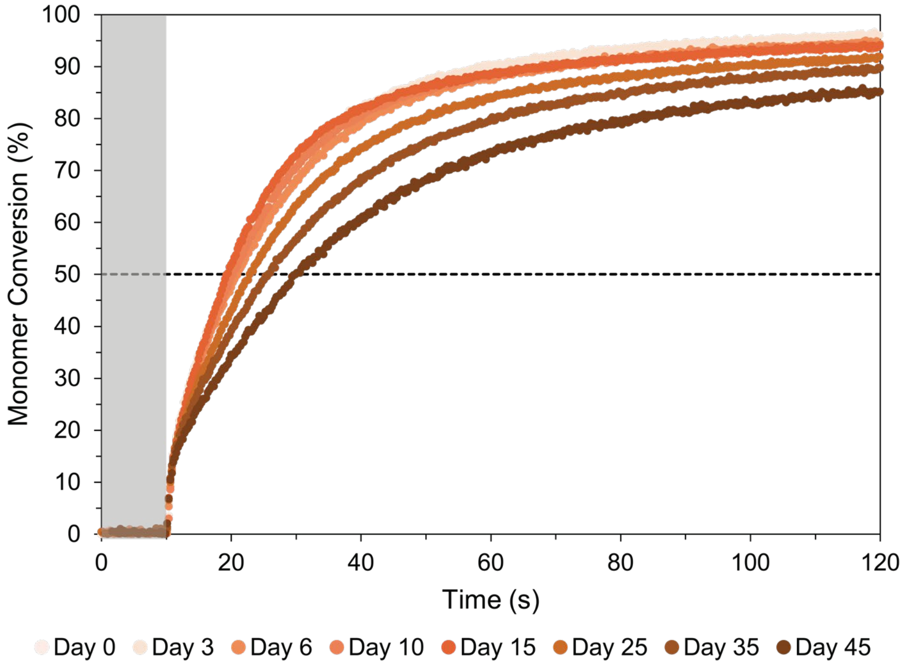

Figure S41. Plot of monomer conversion vs. time for the Type II resin. The samples were degassed before each trial and then irradiated with a 525 nm LED at 20 mW/ cm².

UV/Vis Photodegradation (Ambient)

Optimized resins for both TTA-UC and Type II systems were prepared in a 50/50 wt% mixture of 2-phenoxyethyl acrylate and TMPTA. The resin was loaded between glass slides with 100 µm spacers blanking with the same resin without the **PS** (PtOEP). Samples were then irradiated with a 525 nm LED (10 mW/cm^2) collecting spectra at 1.3 second intervals. A time series of normalized absorbance spectra are shown below.

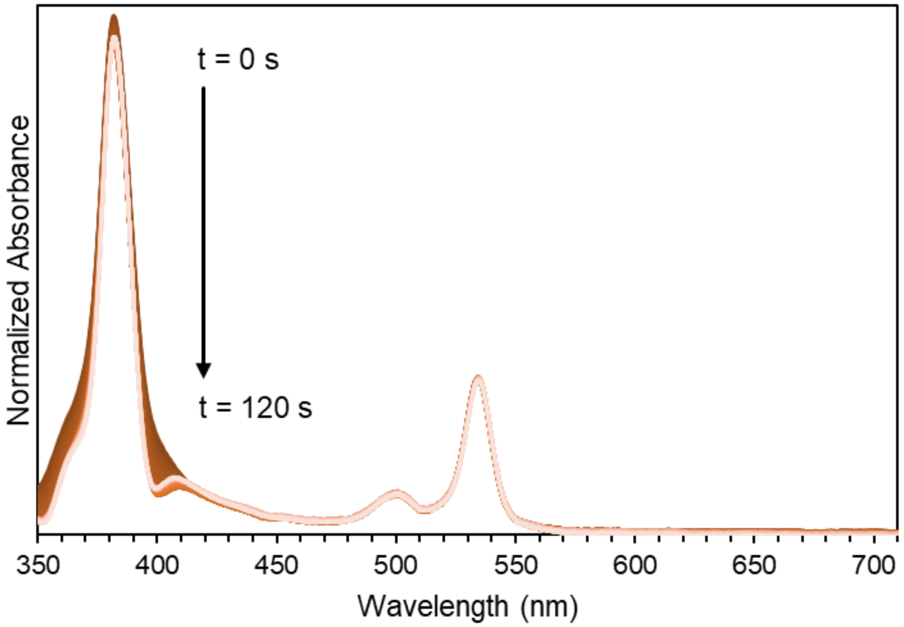

Figure S42. PtOEP absorbance in the TTA-UC resin during 120 s of irradiation with the 525 nm LED. The peak corresponding only to PtOEP absorbance at 535 nm remained constant.

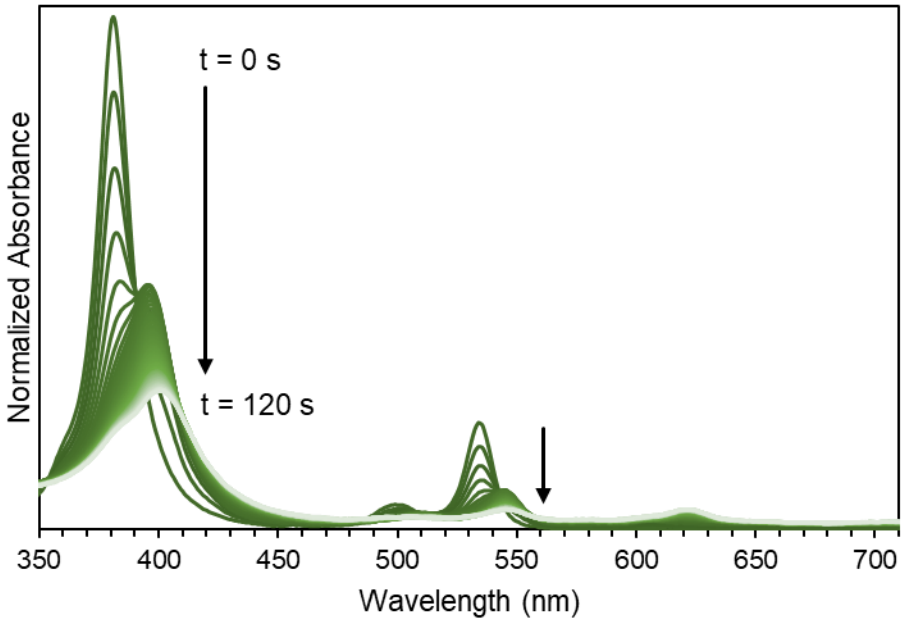

Figure S43. PtOEP absorbance in the Type II resin during 120 s of irradiation with the 525 nm LED.

Transmission FTIR Characterization (Ambient)

Photopolymerization optimization-3 for 3D printing. The effect of BAPO concentration and light intensity on the polymerization kinetics in the presence of the TMPTA crosslinker under ambient conditions were examined via transmission RT-FTIR (**Figures S44-S45**). Threshold intensity was determined under ambient conditions at several intensities between 2 mW/cm^2 and 250 mW/cm^2. The complete data is provided in **Figures 6A** and **6B** in the main manuscript and **Table S8**, with selected intensities plotted in **Figure S46**.

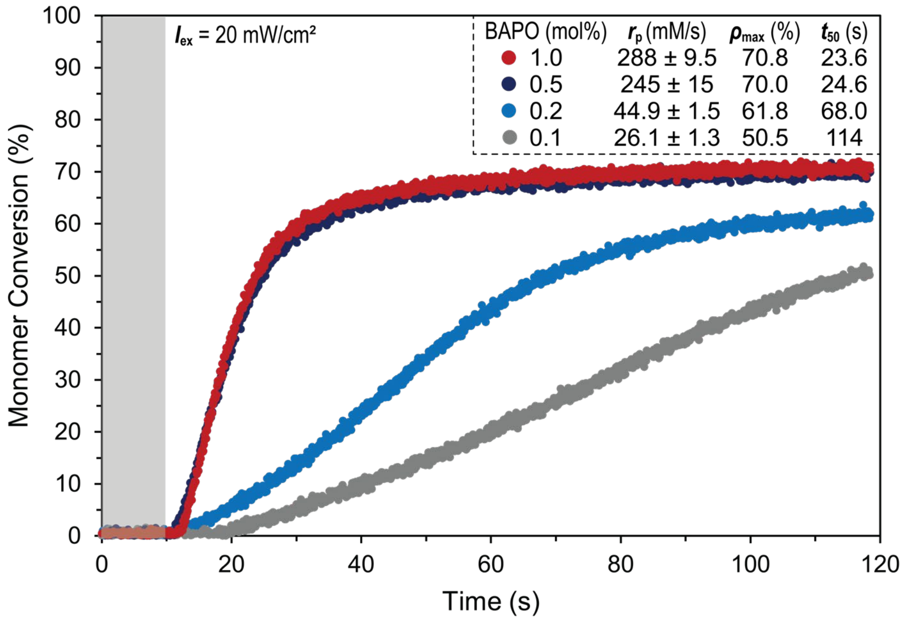

Figure S44. Varying equivalents of BAPO (10, 20, 50, 100) relative to PtOEP in a 50/50 (wt%) mix of 2-phenoxyethyl acrylate and TMPTA loaded between glass slides with 100 µm spacers. Samples irradiated with a 525 nm LED at 20 mW/cm² and measured via transmission FTIR.

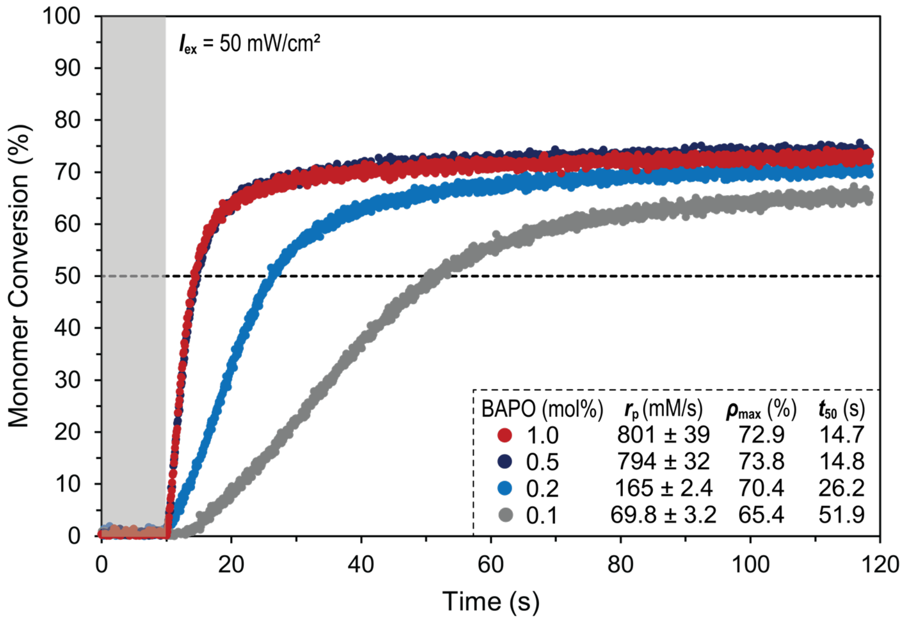

Figure S45. Varying equivalents of BAPO (10, 20, 50, 100) relative to PtOEP in a 50/50 (wt%) mix of 2-phenoxyethyl acrylate and TMPTA loaded between glass slides with 100 µm spacers. Samples irradiated with 525 nm LED at 50 mW/cm² and measured via transmission FTIR.

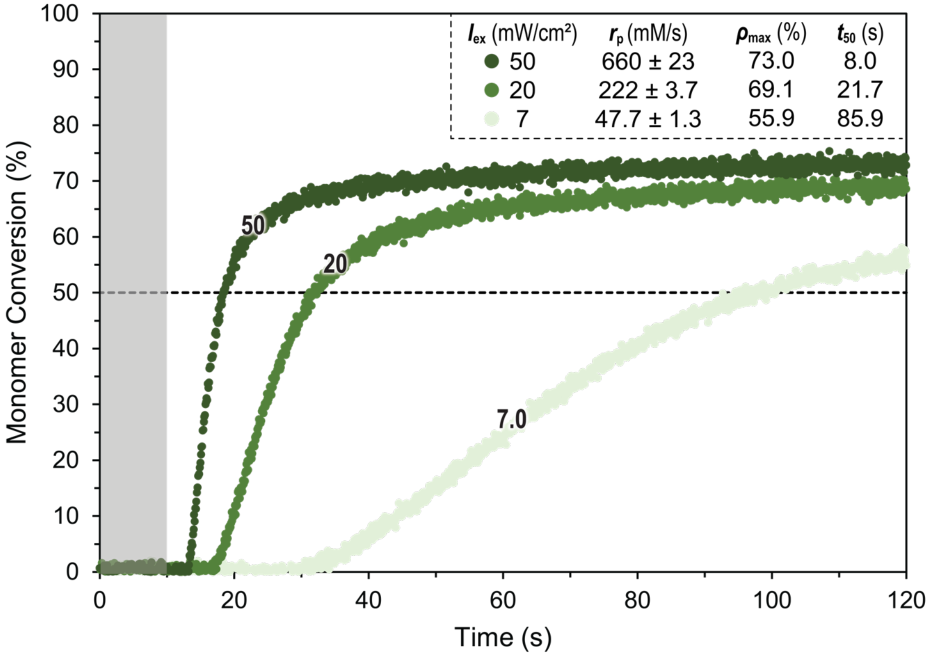

Figure S46. Printing resin - PtOEP (0.01 mol%), DPA (0.10 mol%), and BAPO (0.50 mol%) in a 50/50 (wt%) mix of 2-phenoxyethyl acrylate and TMPTA loaded between glass slides with 100 µm spacers. Samples irradiated with a 525 nm LED (7, 20 and 50 mW/cm²) and measured via transmission FTIR.

Table S8. Summary of TTA-UC to Type I photopolymerization kinetics as a function of light intensity measured using RT-FTIR and provided in Fig. 6A and 6B and Fig. S44. The [PtOEP] was 600 µM and the ratio of PtOEP:DPA:BAPO was 1:10:50. Values are averages from triplicate measurements (or more) with ±1 standard deviation from the mean.

I_{ex} (mW/cm^2)	r_p (mM/s)	ρ_{max} (%)	t_{inh} (s)	t_{50} (s)
2	7.56 ± 0.39	6.2	-	-
5	27.5 ± 0.93	39.5	33.7	-
7	47.7 ± 1.3	55.9	22.0	85.9
10	82.7 ± 1.2	62.9	15.6	53.4
20	222 ± 3.7	69.1	7.0	21.7
50	660 ± 23	73.0	2.9	8.0
100	1412 ± 53	75.7	1.3	3.8
150	2080 ± 160	76.8	0.8	2.5
200	2730 ± 240	77.4	0.6	1.9
250	3220 ± 200	78.1	0.5	1.6

Rheology Studies

First, the gel point was found using an oscillation test (20 mm stainless steel upper plate, 100 µm gap, 1.0 strain%, 10 rad/s angular frequency, torque 10.0 µN•m) irradiating with a 525 nm LED (10 mW/cm^2) starting at 10 s and finding the modulus crossover (**Figure S47**). In a standard cure depth experiment the geometry gap was set to 500 µm. In each run the resin was loaded onto the lower acrylic plate of the photorheology accessory and the upper plate was brought down to a gap height of 500 µm. The LED was triggered to shine through the acrylic plate and into the sample for incrementally shorter times. After each irradiation period, the upper plate was raised and excess monomer gently wicked off of the top of the sample with a Kim-wipe. Then the upper plate was lowered at 1 µm/s while measuring the axial force (N). The cure depth was taken as the gap height at a force of 2 N, which was found to be the force giving the most consistent measure of depth when curing through the 500 µm gap.[S8] The light intensities used were 0.1 mW/cm^2, 0.5 mW/cm^2, and 10 mW/cm^2 for the Type I, Type II, and TTA-UC resins (**Figure S49**), respectively, which were selected because they gave comparable rates of polymerization as determined through previous ATR-FTIR experiments.

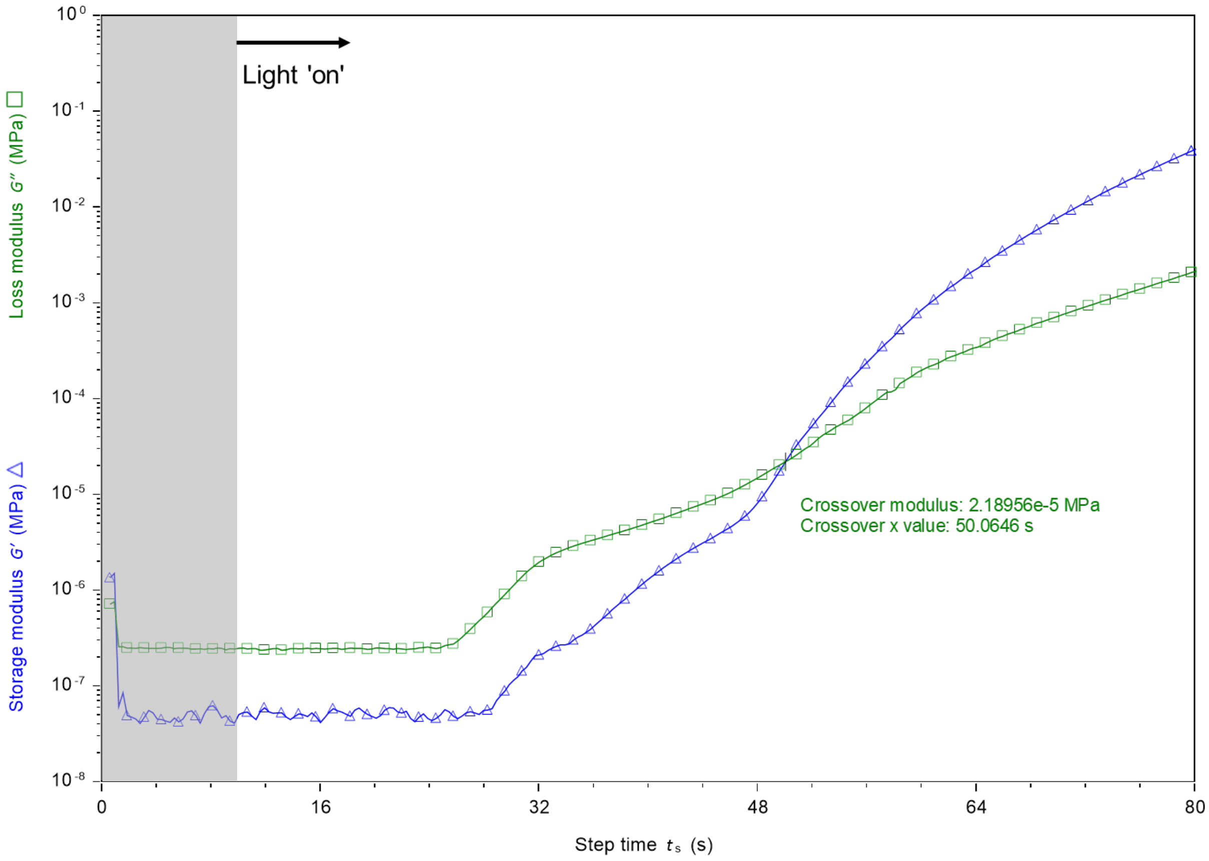

Figure S47. Representative photorheology experiment of the TTA-UC resin to determine the gel point as defined by the crossover in storage and loss moduli. The 525 nm LED (10 mW/cm^2) was turned on at 10 s giving an effective gel point of 39.6 ± 2.4 seconds at a 100 µm gap.

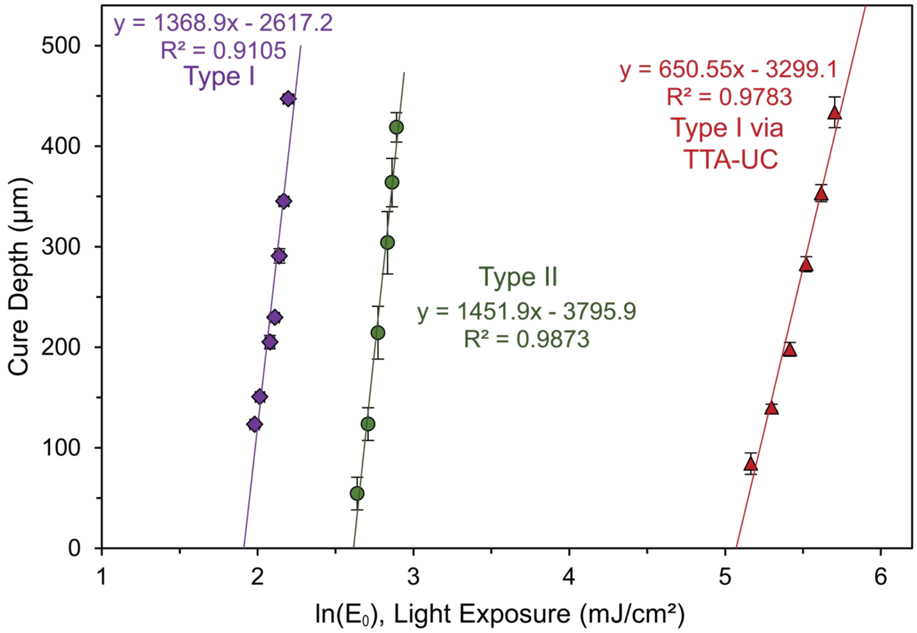

Figure S48. Rheology experiments plotting cure depth (C_d) against the natural log of incident light exposure energy (E_0) used to determine depth of penetration (D_p) as the slope of the linear fit.

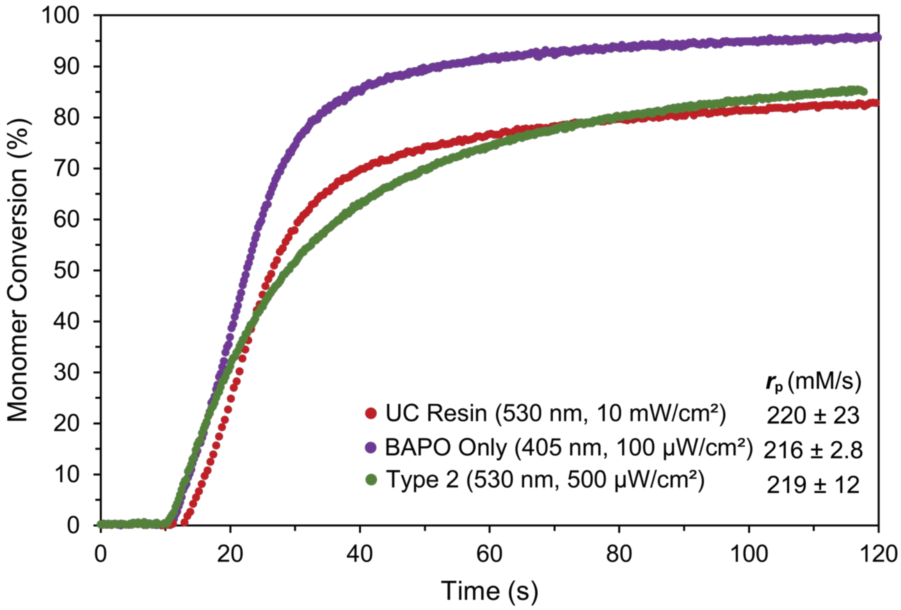

Figure S49. Data collected via FTIR-ATR was used to determine light intensities for the three respective photosystems that provided similar rates of polymerization ~220 mM/s (TTA-UC, 10 mW/cm^2; Type II, 0.5 mW/cm^2; Type I, 0.1 mW/cm^2). These light intensities were used for subsequent photorheology cure depth experiments.

Table S9. Summary of photorheology experiments for TTA-UC, Type I, and Type II as shown in Fig. 6C and Fig. S46-S47. Values are averages from triplicate measurements (or more) with ±1 standard deviation from the mean.

**Mechanism**	$\boldsymbol{E_0}$ **(mJ/cm²)**	$\boldsymbol{C_d}$ **(µM)**
TTA-UC	150	29 ± 9
	175	84 ± 11
	200	140 ± 3
	225	198 ± 7
	250	283 ± 8
	275	353 ± 9
	300	434 ± 15
Type I	7.25	123 ± 3
	7.50	151 ± 2
	8.00	205 ± 7
	8.25	230 ± 3
	8.50	291 ± 7
	8.75	345 ± 4
	9.00	447 ± 4
Type II	12	55 ± 16
	15	124 ± 16
	16	215 ± 26
	17	304 ± 31
	17.5	364 ± 24
	18	419 ± 15

E_0 = incident exposure intensity; C_d = cure depth

Optical Density Matching. The optical densities of the resins over the LED emission range were estimated by determining the overlap between the photoinitiator (BAPO or PtOEP) absorption spectra and the LED emission spectra (**Figures S50** and **S51**). The resulting ratio between the absorbed photons (in a 100 µm thick sample slice) that can initiate polymerization and the total photon flux emitted by the LED was used to calculate the total transmittance of light in the sample. We found a good match between the transmittance when using either BAPO (78%) or PtOEP (79%) and thus we expect the light penetration to be comparable in all three systems. The same overlap was used to quantify ε_{avg} (the average molar absorptivity), following that described previously (**Figure S52**). Based on these results, the predicted D_p values based on equation 4 in the main manuscript for Type I was 920 µm and for the Type II and TTA-UC resins was 892 µm.

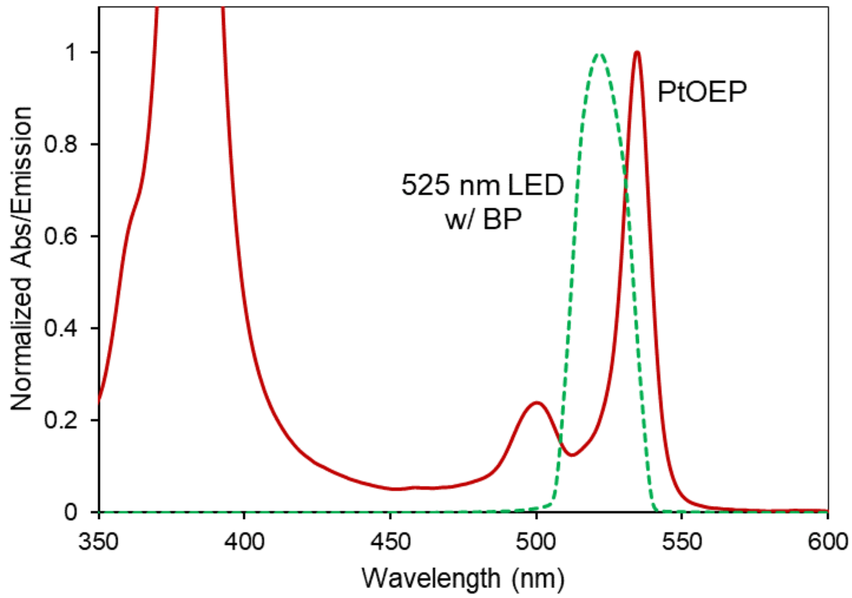

Figure S50. Absorption spectrum of PtOEP (600 µM) in 2-phenoxyethyl acrylate at a thickness of 100 µM overlaid with the emission spectrum of the 525 nm LED with a 525×25 nm bandpass filter. ε_{avg} value provided as an inset.

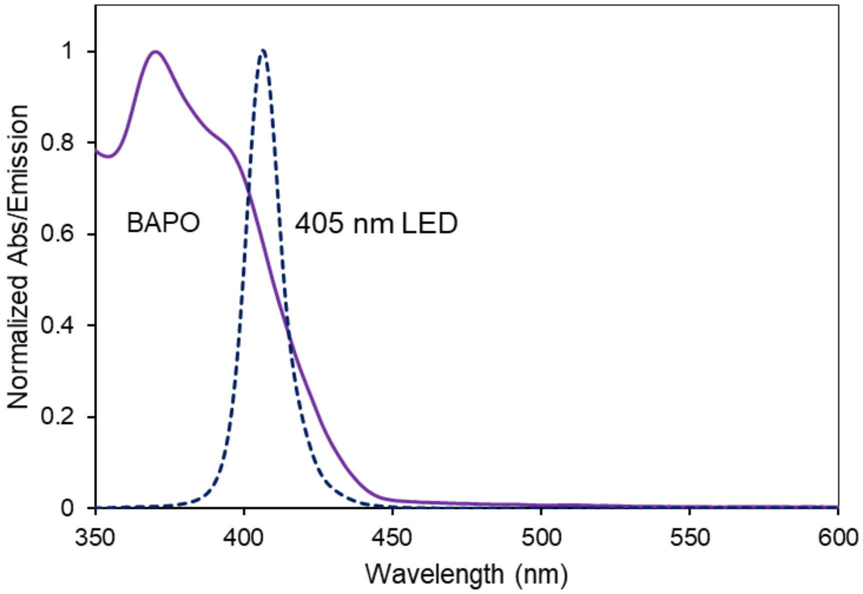

Figure S51. Absorption spectrum of BAPO (30 mM) in 2-phenoxyethyl acrylate at a thickness of 100 µM overlaid with the emission spectrum of the 405 nm LED.

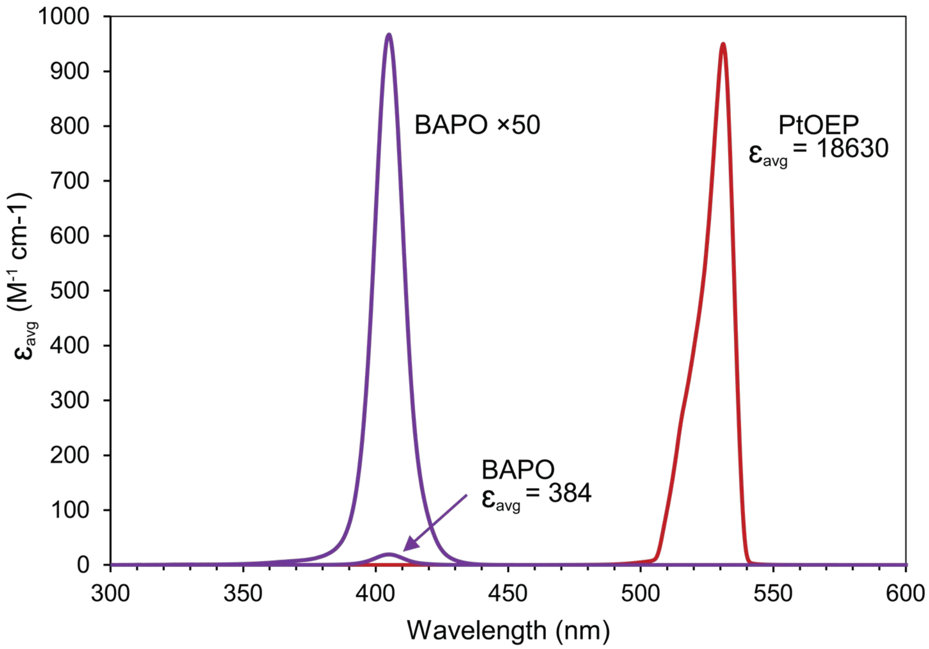

Figure S52. Average extinction spectrum based on the overlap of either BAPO or PtOEP with the 405 nm and 525 nm LED, respectively. ε_{avg} value calculated as the integral of the trace.

3D Printing

Time-based 3D printing array for exposure time optimization. The below resolution print was done at a layer thickness of 100 µm using a custom 1-channel DLP 3D printer with a 525 nm LED (**Figure S53**) to qualitatively identify the optimal exposure time. Each resolution print contains a set of squares (5 – 115 sec in 10 sec increments) varying exposure time/layer (**Figures S54a** and **S54b**).

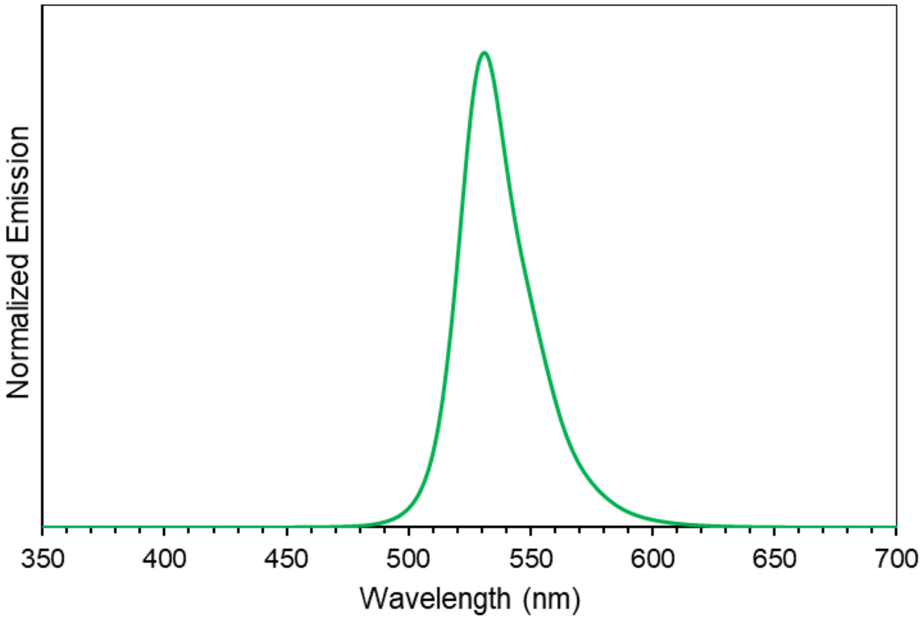

Figure S53. Emission profile of the Green LED (525 nm) in the DLP 3D printer

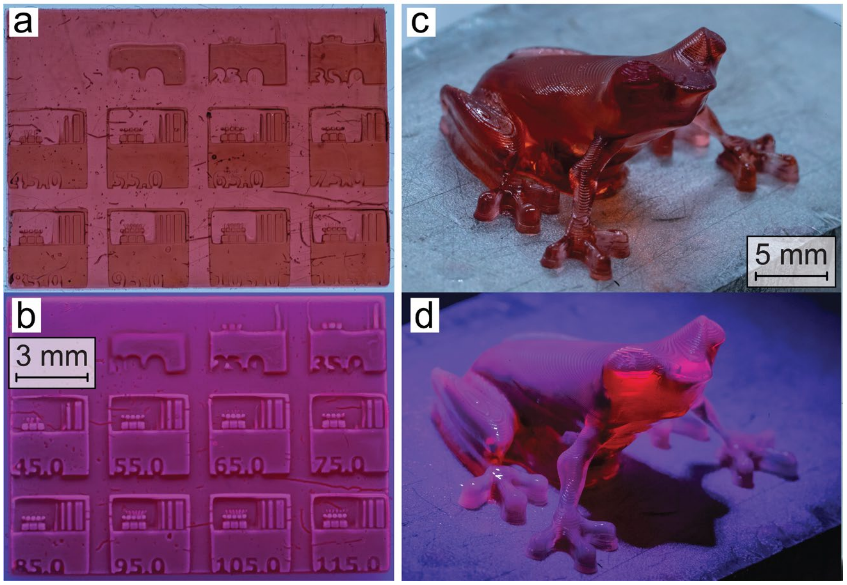

Figure S54. 3D prints using TTA-UC system. (a) Resolution print under ambient light and (b) phosphorescing under UV (365 nm) light. (c) Frog 3D print under ambient and (d) UV (365 nm) light.

Video Captions

Video S1. Spectral evolution of the TTA-UC photopolymerization without photoinitiator. The sample was prepared in 2-phenoxyethyl acrylate using the same concentrations as in the upconversion and FTIR studies (0.6 mM of PtOEP and 6 mM of DPA) in a 1 mm pathlength cuvette without any photoinitiator and purged with nitrogen gas. The spectra and video were collected at the same time under 532 nm laser excitation at 200 mW/cm^2 for 6 minutes. The spectra were collected from the front-face of the cuvette using the above described upconversion setup, with 100 ms integration time and averaging 5 spectra in the spectrometer resulting in two spectra per second being transferred and saved on a computer. The individual spectra were collated as a video in MATLAB. The video was collected behind the cuvette to reduce laser scatter with a Sony A7SII camera and Sony FE 2.8/90 G OSS Macro Lens. The video is played at 10× speed.

Video S2. Side-by-side comparison of emission from TTA-UC printing resin during transmission-FTIR. Two samples were prepared in a 50/50 wt% mixture of 2-phenoxyethyl acrylate and TMPTA using the standard concentrations (0.6 mM of PtOEP and 6 mM of DPA) either without BAPO (shown left), or with 30 mM BAPO present (shown right). FTIR spectra and video were collected with the sample loaded between glass slides at a 100 µm spacing under ambient conditions. Both are irradiated with the 525 nm LED (100 mW/cm^2) equipped with a 525×25 nm bandpass filter. The video was taken through a 500 nm shortpass filter (Edmund Optics, #15-257) using a Sony A7SII camera and Sony FE 2.8/90 G OSS Macro Lens. The video is played in real time (1× speed).

REFERENCES

(S1) Ahn, D.; Stevens, L. M.; Zhou, K.; Page, Z. A. Rapid High-Resolution Visible Light 3D Printing. *ACS Cent. Sci.* **2020**. https://doi.org/10.1021/acscentsci.0c00929.

(S2) Mongin, C.; Golden, J. H.; Castellano, F. N. Liquid PEG Polymers Containing Antioxidants: A Versatile Platform for Studying Oxygen-Sensitive Photochemical Processes. *ACS Appl. Mater. Interfaces* **2016**, *8* (36), 24038–24048. https://doi.org/10.1021/acsami.6b05697.

(S3) Kubin, R. F.; Fletcher, A. N. Fluorescence Quantum Yields of Some Rhodamine Dyes. *J. Lumin.* **1982**, *27* (4), 455–462. https://doi.org/10.1016/0022-2313(82)90045-X.

(S4) Yanai, N.; Suzuki, K.; Ogawa, T.; Sasaki, Y.; Harada, N.; Kimizuka, N. Absolute Method to Certify Quantum Yields of Photon Upconversion via Triplet-Triplet Annihilation. *J. Phys. Chem. A* **2019**, *123* (46), 10197–10203. https://doi.org/10.1021/acs.jpca.9b08636.

(S5) Aulin, Y. V.; Van Sebille, M.; Moes, M.; Grozema, F. C. Photochemical Upconversion in Metal-Based Octaethyl Porphyrin-Diphenylanthracene Systems. *RSC Adv.* **2015**, *5* (130), 107896–107903. https://doi.org/10.1039/c5ra20602b.

(S6) Bachilo, S. M.; Weisman, R. B. Determination of Triplet Quantum Yields from Triplet−Triplet Annihilation Fluorescence. *J. Phys. Chem. A* **2000**, *104* (33), 7711–7714. https://doi.org/10.1021/jp001877n.

(S7) Cheng, Y. Y.; Fückel, B.; Khoury, T.; Clady, R. G. C. R.; Tayebjee, M. J. Y.; Ekins-Daukes, N. J.; Crossley, M. J.; Schmidt, T. W. Kinetic Analysis of Photochemical Upconversion by Triplet-Triplet Annihilation: Beyond Any Spin Statistical Limit. *J. Phys. Chem. Lett.* **2010**, *1* (12), 1795–1799. https://doi.org/10.1021/jz100566u.

(S8) Rau, D. A.; Reynolds, J. P.; Bryant, J. S.; Bortner, M. J.; Williams, C. B. A Rheological Approach for Measuring Cure Depth of Filled and Unfilled Photopolymers at Additive Manufacturing Relevant Length Scales. *Addit. Manuf.* **2022**, *60* (A), 103207. https://doi.org/10.1016/j.addma.2022.103207.
